# Emotional Distress Statistically Accounts for the Cross-Sectional Association Between Spiritual Well-Being and Global Quality of Life in Patients with Cancer

**DOI:** 10.3390/healthcare14193209

**Published:** 2026-09-28

**Authors:** Dana Sonia Nagy, Vlad-Norin Vornicu, Alina-Gabriela Negru, Serban Mircea Negru

**Affiliations:** 1 Doctoral School, “Victor Babes” University of Medicine and Pharmacy, Eftimie Murgu Square No. 2, 300041 Timisoara, Romania; 2 Department of Oncology, “Victor Babes” University of Medicine and Pharmacy, Bd. Victor Babes No. 16, 300226 Timisoara, Romania; vlad.vornicu@umft.ro (V.-N.V.); serban.negru@umft.ro (S.M.N.); 3 Department of Palliative Care, OncoHelp Hospital Timisoara, Ciprian Porumbescu Street No. 59, 300239 Timisoara, Romania; 4 Department of Cardiology, “Victor Babes” University of Medicine and Pharmacy, 300041 Timisoara, Romania; alinanegru@umft.ro

**Keywords:** neoplasms, spirituality, quality of life, anxiety, palliative care

## Abstract

Background and Objectives: Spiritual well-being is consistently associated with better quality of life in oncology, but the pathways involved are disputed. Two competing accounts exist: spiritual well-being may buffer patients against the impact of physical symptoms, or it may act mainly through emotional distress. We examined whether cross-sectional data from a Romanian cancer cohort are compatible with either account. Materials and Methods: In this single-centre cross-sectional study, 175 adults with cancer completed the FACIT-Sp-12, the Religious Commitment Inventory-10, the EORTC QLQ-C30 and the Hospital Anxiety and Depression Scale; the FACIT-Sp-12, QLQ-C30 and HADS were administered in locally adapted formats that deviate from the validated instruments, so all findings pertain to these adapted versions. Patients were grouped by tertile of FACIT-Sp-12 score. The primary outcome was global health status/quality of life. Multivariable linear regression with heteroscedasticity-consistent standard errors, prespecified bootstrapped mediation analyses (interpreted as cross-sectional statistical decompositions), a multiplicative moderation test, restricted cubic splines and nine prespecified sensitivity analyses were performed. Results: Patients were 62.2 ± 13.1 years old, 65.1% had advanced disease and 51.4% screened positive for clinically significant distress (HADS-Anxiety or HADS-Depression ≥ 8). Global quality of life rose monotonically across spiritual well-being tertiles (53.8 ± 28.0, 65.9 ± 22.3 and 74.5 ± 23.1 points; *p* < 0.001), with an adjusted high-versus-low difference of 14.9 (4.3 to 25.5) points. In the prespecified primary model, each standard deviation of spiritual well-being was associated with 7.15 (3.45 to 10.86) points of global quality of life. In separate prespecified single-mediator models, the indirect associations through HADS-anxiety (4.21 points, 95% CI 1.89 to 6.86) and HADS-depression (3.03 points, 95% CI 1.17 to 5.43) were both statistically significant (bootstrapped *p* < 0.001); these cross-sectional decompositions are equally compatible with reverse, bidirectional or common-cause structures. No statistically detectable spiritual well-being × symptom burden interaction was found (β = −1.198, 95% CI −6.355 to 3.959; *p* = 0.649), but the confidence interval included clinically relevant effect modification in either direction, and there was no evidence of departure from linearity in the dose–response relationship (*p* for non-linearity = 0.510). Coefficients for the peace subscale were consistently larger than those for faith, although the subscales were modelled separately. Conclusions: Higher scores on the adapted FACIT-Sp-12 were cross-sectionally associated with better global quality of life and lower emotional distress. Anxiety and depression statistically accounted for part of this association, but temporal ordering and causality cannot be determined from these data, and the interaction analysis was too imprecise to support or exclude buffering. Routine assessment of spiritual and emotional concerns alongside symptom control is supported; whether interventions that increase spiritual well-being improve quality of life requires longitudinal and interventional study using the validated instrument formats.

## 1. Introduction

Cancer remains one of the leading causes of premature death worldwide, with an estimated 20 million new cases and 9.7 million deaths recorded in 2022 [1]. Europe carries a disproportionate share of this burden, accounting for roughly a quarter of global cancer cases despite hosting less than a tenth of the world population [2]. Within Europe, Romania and the other countries of the eastern periphery display a distinctive epidemiological profile characterised by later stage at presentation, lower screening uptake, pronounced rural–urban inequalities and cancer mortality rates that have declined more slowly than the continental average, a pattern documented across cancer sites in the EU Country Cancer Profile for Romania [3] and, for breast cancer specifically, in national mortality analyses [4]; rural–urban survival inequalities have also been described in Romanian registry data [5]. A consequence of this pattern is that a large proportion of patients enter oncology services with advanced or metastatic disease, for whom the realistic therapeutic goal is not cure but the preservation of function, symptom control and quality of life. In such populations, patient-reported outcomes are not ancillary endpoints but the principal measures of benefit.

Alongside physical and psychological domains, spirituality is now formally recognised as a constitutive dimension of comprehensive cancer and palliative care. The 2009 Consensus Conference defined spiritual care as an essential component of quality palliative care and established the framework for spiritual screening, history-taking and assessment that remains in use today [6]. A subsequent systematic evidence review, drawing on hundreds of studies in serious illness, concluded that spirituality is a salient patient-reported dimension of health that clinicians should address directly rather than delegate by default [7]. Professional guidance has followed the evidence: the American Society of Clinical Oncology, in its most recent guideline update, recommends early integration of palliative care, explicitly including attention to spiritual and existential concerns, into standard oncology practice for patients with advanced disease [8]. What remains far less settled is exactly how spiritual resources translate into better patient-reported outcomes.

The empirical association itself is well replicated. A systematic review of scale- and factor-level analyses found spiritual well-being to be positively associated with quality of life across a wide range of cancer populations and instruments, with the strongest and most consistent effects arising from the existential components of spirituality rather than from religious belief per se [9]. A large meta-analytic synthesis similarly demonstrated that religion and spirituality correlate modestly but reliably with better mental health in patients with cancer, again with the affective and existential dimensions carrying most of the signal [10]. These convergent findings have motivated interest in spirituality as a modifiable target for supportive interventions rather than merely as a descriptive characteristic of patients.

Two explanatory accounts are commonly invoked to explain this association, and they carry markedly different clinical implications. The first is a stress-buffering account: patients with greater spiritual resources are held to be less affected, point for point, by any given level of physical symptom burden, so that the slope relating symptoms to quality of life is flattened. If correct, spiritual resources would be expected to matter most where symptom control is incomplete; even so, spiritual care would remain a complementary component of multidimensional supportive care rather than an alternative to symptom management. The second is a mediation account: spiritual well-being is held to be associated with lower anxiety and depressive symptoms, which are in turn associated with worse global quality of life, so that any association is additive to, and independent of, symptom burden. The two accounts are rarely tested against one another in the same cohort, and unmet spiritual needs remain common and independently associated with worse emotional well-being irrespective of which mechanism operates [11]. It is important to distinguish the causal mediation hypothesis implied by the second account from what a cross-sectional dataset can test. With exposure, putative mediators and outcome measured at a single time point, a statistical indirect-association model can establish whether the data are compatible with the hypothesised pathways, but it cannot adjudicate between causal mechanisms or exclude reverse or bidirectional pathways [12].

Evidence from Eastern Europe is particularly scarce, and this matters more than it may appear. Most published cohorts derive from Western European, North American or East Asian settings in which religious affiliation is heterogeneous and institutional religious practice is comparatively low. Romania represents an almost inverted context: in the 2021 national census, 85% of respondents who declared a religion identified as Orthodox and fewer than 1% reported no religion [13]; levels of religious belief and practice are among the highest in Europe [14]; and intensification of religious practice during illness is commonly reported by Romanian patients [15], a pattern also observed in the present cohort (Table 1). Such a setting provides unusual statistical leverage for separating the existential dimensions of spirituality, which vary considerably between individuals, from religious commitment, which is close to a cultural constant. Where religiosity is nearly universal, any residual association between quality of life and the existential components of spiritual well-being cannot be attributed simply to belonging to a religious community.

We therefore designed the present study to examine whether cross-sectional data are compatible with the buffering account, the mediation account, or both, in a consecutively recruited Romanian cancer cohort managed across oncology and palliative care services. Our primary objective was to quantify the association between spiritual well-being and global health status/quality of life, and to examine whether that association is statistically accounted for by emotional distress, whether it is modified by physical symptom burden, or both. Secondary objectives were to characterise the shape of the dose–response relationship, to compare the relative contributions of the meaning, peace and faith dimensions of spiritual well-being and of formal religious commitment across the full range of functional and symptom domains, and to establish the robustness of all estimates under a comprehensive set of prespecified sensitivity analyses.

The following hypotheses and analytic hierarchy were specified before the data were examined. The primary hypothesis (H1) was that the FACIT-Sp-12 total score would be positively associated with global health status/quality of life after adjustment for age, sex, advanced disease, ECOG performance status and comorbidity count; the primary estimand is the covariate-adjusted linear regression coefficient per one standard deviation of FACIT-Sp-12, estimated with HC3 standard errors (Model 1, Section 2.4). Three secondary confirmatory hypotheses, each tested once, were specified alongside it: that HADS-anxiety would statistically account for part of this association (H2a, indirect association through anxiety); that HADS-depression would do so (H2b, indirect association through depression); and that composite symptom burden would modify the association (H3, multiplicative interaction). The confirmatory analysis therefore comprised four prespecified tests (H1, H2a, H2b and H3); the multiplicity approach is described in Section 2.4. All other analyses planned before the data were examined—the tertile contrasts, subgroup analyses, subscale- and domain-specific models, the three additional single-mediator pathways in Section 3.8, the spline analysis and the nine sensitivity analyses—are prespecified exploratory analyses. Four analyses were introduced during peer review and were not part of the original analysis plan: the parallel multiple-mediator model (Section 3.8), the mutually adjusted subscale model with Wald tests of between-subscale differences (Section 3.5), the interaction between Orthodox affiliation and spiritual well-being (Section 4.1) and a sensitivity analysis in which the peace subscale was removed from the exposure to gauge the contribution of affective item content shared with the HADS (Section 3.8); these are labelled as post hoc wherever they appear.

## 2. Materials and Methods

### 2.1. Study Design, Setting and Population

This was a single-centre, cross-sectional, observational study conducted in the oncology and palliative care services of OncoHelp Hospital, Timisoara, Romania (the medical oncology outpatient clinic and day hospital, and the palliative care inpatient unit and outpatient clinic). Recruitment took place between January 2025 and September 2025. Consecutive adult patients attending for systemic anticancer treatment, follow-up or supportive and palliative care were invited to participate during routine clinical contacts. The study was designed and reported in accordance with the STROBE recommendations for observational research. Because the exposures of interest are patient-reported psychological and existential constructs rather than biological measurements, all data were collected through a single structured, interviewer-supported questionnaire administered in Romanian at a single time point, with clinical variables abstracted contemporaneously from the medical record. No follow-up assessments were performed, and no intervention was delivered as part of the study.

Participant flow was documented as follows. During the recruitment period, 214 patients were assessed for eligibility; 17 did not meet the inclusion criteria (documented cognitive impairment, delirium or severe psychiatric illness, *n* = 8; not aware of the diagnosis, *n* = 5; unable to complete a questionnaire in Romanian, *n* = 4); 12 were judged by the treating clinician to be too unwell for questionnaire completion; and 10 declined participation. The remaining 175 patients (81.8% of those assessed) consented, completed the assessment battery and were analysed; no consenting patient was subsequently excluded.

The final analytic sample comprised 175 patients. No a priori sample-size calculation was performed; the sample size was determined by the number of eligible patients recruited during the enrolment period. We therefore make no claim that the study was adequately powered for the mediation, interaction, spline or subgroup analyses, and the precision of those estimates is conveyed by their confidence intervals. As a post hoc indication of the precision available for the interaction test, the smallest interaction detectable with 80% power at α = 0.05 is derived from the standard error of the fitted interaction term and reported in Section 4.1. The study was approved by the Institutional Ethics Committee of the “Victor Babes” University of Medicine and Pharmacy, Timisoara (approval no. 47/19.12.2024, dated 19 December 2024), was conducted in accordance with the Declaration of Helsinki, and written informed consent was obtained from every participant before any study procedure. Questionnaires were pseudonymised at entry and the analysis dataset contained no direct identifiers.

### 2.2. Eligibility Criteria and Group Allocation

Eligible patients were aged 18 years or older, had a histologically or cytologically confirmed malignant neoplasm of any primary site and any stage, were aware of their diagnosis, and were able to give informed consent and to complete a questionnaire in Romanian. Patients were excluded if they had documented cognitive impairment, delirium or a psychiatric condition severe enough to preclude reliable self-report, if their clinical condition made questionnaire completion burdensome in the judgement of the treating clinician, or if they declined participation. All disease stages were deliberately admitted, including patients in remission, because restricting recruitment to advanced disease would have truncated the range of both symptom burden and quality of life and thereby attenuated the very associations under study.

This is an observational study without an intervention, so there was no randomised allocation. For the group-wise analyses, patients were classified into three ordered exposure groups defined by tertiles of the FACIT-Sp-12 total score, computed within the study sample: a low spiritual well-being group (≤37 points; *n* = 59), an intermediate group (>37 to ≤44 points; *n* = 66) and a high group (>44 points; *n* = 50). The low tertile served as the reference comparison group throughout. Tertiles were chosen a priori over a median split so that a monotonic dose–response pattern could be inspected while retaining adequate cell sizes; the grouping was used for descriptive and comparative purposes only, and all inferential analyses of the primary hypothesis retained spiritual well-being on its original continuous scale to avoid the loss of information and the residual confounding that categorisation introduces.

### 2.3. Variables, Outcomes and Data Collection

Spiritual well-being was measured with a locally adapted version of the 12-item Functional Assessment of Chronic Illness Therapy—Spiritual Well-Being scale (FACIT-Sp-12) [16]. The Romanian-language items were the licenced FACIT.org translation, used with permission. However, the locally printed questionnaire presented a four-point response format (1 = not at all, 2 = somewhat, 3 = quite a bit, 4 = very much) instead of the validated five-point 0–4 format, so that the total score ranges from 12 to 48 and each subscale from 4 to 16 rather than 0–48 and 0–16. Scores are therefore not directly comparable with published norms, and all analyses use the scale on its original continuous metric or as within-sample tertiles. Items 4 and 8 were reverse-keyed before scoring. Because no psychometric evaluation of this response format was available, its properties were examined in the present sample (see the final paragraph of this section). In addition to the total score, three subscale scores were derived following the validated three-factor solution—meaning (items 2, 3, 5 and 8), peace (items 1, 4, 6 and 7) and faith (items 9–12)—because the meaning/peace and faith components are known to behave differently against psychological outcomes [17]. Formal religious commitment was assessed with the Religious Commitment Inventory-10 (RCI-10), a 10-item measure of the behavioural and cognitive salience of religion, scored on a five-point scale (1 = not at all true of me to 5 = totally true of me), so that the 10-item total ranges from 10 to 50 [18]. No validated Romanian version of the RCI-10 exists; the items were forward- and back-translated by two bilingual clinicians and reconciled by the study team. The paper form recorded the 10-item total score, which was used in all analyses. Every recorded total was re-verified against the original paper forms: all 175 totals lay within the attainable 10–50 range (observed range 10–46), and no alternative coding was used. During data entry, item-level responses were transcribed for eight of the ten items only; items 2 (financial contributions to one’s religious organisation) and 10 (being informed about and influential in one’s local religious group) were not retained at item level. Consequently, the reliability coefficient reported below refers to the eight transcribed items, and the accuracy of the recorded 10-item total could be verified only indirectly: the eight-item sum correlated at r = 0.98 with the recorded total, and substituting the eight-item sum (rescaled to the 10-item metric) for the recorded total in the subscale-by-outcome regression models changed no coefficient by more than 0.3 points. Principal-axis factoring of the eight transcribed items yielded a single dominant factor (first eigenvalue 5.4; all loadings ≥ 0.66), consistent with the unidimensional structure of the original instrument. Additional single-item spirituality variables recorded religious affiliation, frequency of attendance at religious services, the self-rated importance of faith, whether the patient had sought spiritual support during the illness, and whether illness had changed religious practices or beliefs.

Quality of life was measured with the European Organisation for Research and Treatment of Cancer QLQ-C30 [19], administered in a locally adapted format described below. Scale scores for the global health status/quality of life item pair, the five functional scales and the symptom scales and single items were computed by linear transformation to a 0–100 metric according to the standard EORTC algorithm, with higher functional and global scores denoting better status and higher symptom scores denoting greater burden. The Romanian version of the QLQ-C30 supplied by the EORTC Quality of Life Group was used under the standard user agreement; however, the locally printed form deviated from the standard instrument in two respects, which are disclosed in full here. First, the symptom items 8–19 and 28 were printed with a five-point frequency format (1 = never, 2 = rarely, 3 = sometimes, 4 = often, 5 = always) instead of the standard four-point intensity format (1 = not at all, 2 = a little, 3 = quite a bit, 4 = very much). These responses were linearly rescaled to the 1–4 metric before the EORTC algorithm was applied (rescaled value = 1 + 0.75 × (response − 1), so that responses of 1, 2, 3, 4 and 5 became 1, 1.75, 2.5, 3.25 and 4, respectively). The functional items 6–7 and 20–27 and the two global health status/quality-of-life items 29 and 30 retained the standard response formats and were unaffected. Second, physical functioning item 5 (needing help with eating, dressing, washing or using the toilet) was absent from the local form; the physical functioning scale was therefore computed from items 1–4 and is referred to throughout as an adapted four-item physical functioning score, which has not been validated against the standard five-item scale and should be interpreted with that caveat. A scale score was computed when at least half of its constituent items were answered. A composite symptom burden score was defined a priori as the unweighted mean of the eight physical symptom scales and items of the QLQ-C30 (fatigue, nausea/vomiting, pain, dyspnoea, insomnia, appetite loss, constipation and diarrhoea), and a functional summary score as the unweighted mean of the five functional scales. Neither is a standard EORTC score. The financial difficulties item was excluded from the symptom composite because it measures economic rather than physical burden; equal weighting follows the approach of the validated QLQ-C30 summary score, in which scales are combined without differential weights [20]. The composite was intended as a parsimonious index of overall physical symptom load for the moderation analysis, not as a substitute for the domain-specific scores, which are reported in full. Psychological distress was measured with the Hospital Anxiety and Depression Scale, yielding separate 7-item anxiety and depression subscales scored 0–21, with the conventional threshold of 8 or more points denoting a clinically significant case [21]. Clinically significant distress in this report is defined as a score of 8 or more on either subscale. The validated Romanian translation of the HADS was used under licence. Sociodemographic variables (age, sex, residence, education, marital status) and clinical variables (primary tumour site, disease status, ECOG performance status, number of non-oncological comorbidities, time since diagnosis and treatment modalities received) were recorded concurrently. The primary outcome was the EORTC QLQ-C30 global health status/quality of life score. Secondary outcomes were the functional summary score, the composite symptom burden, emotional functioning, and the HADS anxiety and depression scores.

Internal consistency was acceptable to excellent for every instrument in this sample: Cronbach’s α was 0.879 for the FACIT-Sp-12 total (0.727 for meaning, 0.765 for peace and 0.887 for faith), 0.931 for the eight transcribed RCI-10 items (items 2 and 10 were not available at item level, as described above), 0.859 for HADS-anxiety, 0.778 for HADS-depression and 0.905 for the pooled EORTC functional items. Three HADS items had been entered against the inverted response column of the local questionnaire: HADS-depression items 4 (“I can laugh and see the funny side of things”) and 6 (“I feel cheerful”) and HADS-anxiety item 7 (“I can sit at ease and feel relaxed”). On the authorised instrument the response options for these items are printed in descending order of severity (0 = definitely as much/most of the time … 3 = not at all), whereas the local form printed them in ascending order, so the entered codes were reflected before analysis (0 was recoded as 3, 1 as 2, 2 as 1 and 3 as 0). The error was identified during routine data cleaning, before any exposure–outcome association had been examined, when the three items showed negative corrected item–total correlations; the correction was verified against the wording and response order of the authorised Romanian instrument. After re-keying, all corrected item–total correlations were positive (0.41–0.72 for anxiety; 0.38–0.64 for depression) and subscale reliability rose to the values reported above.

Because the FACIT-Sp-12 was administered on a non-standard response format, its structure was examined in the present sample. Principal-axis factoring with oblique rotation reproduced the three-factor meaning–peace–faith solution, with all twelve items loading ≥ 0.40 on their intended factor and no cross-loading above 0.30; inter-factor correlations were 0.61 (meaning–peace), 0.40 (meaning–faith) and 0.37 (peace–faith), in line with the observed subscale correlations. Floor and ceiling effects were quantified as the proportion of participants at the minimum and maximum attainable scores. No participant scored at the floor of any FACIT-Sp subscale. Ceiling effects were present for faith (34.3%; 60/175), meaning (28.6%; 50/175) and peace (19.4%; 34/175) and were concentrated in the high tertile (*n* = 50), in which 42 (84.0%), 40 (80.0%) and 28 (56.0%) patients, respectively, scored at the subscale maximum; 12 patients (6.9% of 175), all in the high tertile, scored at the maximum of the total scale. These counts are mutually consistent: of the 50 patients in the high tertile, 12 scored at the ceiling of all three subscales (and therefore of the total scale), 36 at the ceiling of exactly two subscales and 2 at the ceiling of one. For the primary outcome, 8.6% (15/175) scored 100 and 1.1% (2/175) scored 0. The consequences of this restricted range for the regression, spline, subgroup and subscale analyses are considered in the Discussion.

Two points about these psychometric checks should be made explicit. First, internal consistency, factor structure and the absence of floor effects describe the behaviour of the adapted instruments within this sample; they do not establish measurement equivalence with the validated FACIT-Sp-12, EORTC QLQ-C30 or HADS, which would require formal invariance testing against the standard formats in an independent sample. Second, for this reason, the FACIT-Sp-12, the QLQ-C30 symptom and physical functioning scales and the HADS are referred to throughout as locally adapted versions, and every estimate reported below pertains to those adapted versions rather than to the original instruments. Absolute scores and effect sizes should therefore not be compared directly with published norms or with studies that used the standard formats, whereas the within-sample associations and covariate-adjusted coefficients, which are the object of this study, are interpretable on their own terms.

### 2.4. Statistical Analysis

Continuous variables are summarised as mean ± standard deviation, or as median with interquartile range where the distribution was skewed or the variable ordinal; categorical variables are summarised as counts with column percentages. Comparisons across the three spiritual well-being tertiles used one-way analysis of variance for approximately symmetric continuous variables, the Kruskal–Wallis test for ordinal and skewed variables, and Pearson’s χ^2^ test for categorical variables, or the Fisher–Freeman–Halton exact test (Monte Carlo approximation, 10,000 samples) where any expected cell count was below five. Because several symptom scales are bounded and skewed with floor effects, the between-tertile comparisons of symptom and functional scales were repeated with Kruskal–Wallis tests as a check on the analysis-of-variance assumptions. Effect sizes are reported alongside every significance test: η^2^ for analysis of variance, ε^2^ (epsilon-squared) for Kruskal–Wallis tests, Cramér’s V for contingency tables, and Hedges’ g with its 95% confidence interval for pairwise mean differences. Normality was assessed by inspection of residual quantile plots and homoscedasticity by residual-versus-fitted plots; because mild heteroscedasticity was evident for the primary outcome, all linear models were fitted with HC3 heteroscedasticity-consistent standard errors, which are robust to non-constant variance without requiring transformation of the outcome. Welch’s correction was applied to two-group mean comparisons.

Associations between spirituality measures and outcomes were estimated with multivariable linear regression using a prespecified hierarchy of three models, with covariates selected on clinical grounds rather than by stepwise procedures. Model 1 (primary) adjusts for the baseline confounders age, sex, advanced disease status, ECOG performance status and comorbidity count and yields the primary estimand: the total covariate-adjusted association between FACIT-Sp-12 and global quality of life. Model 2 adds composite symptom burden, treated as a competing explanatory variable that may precede both spiritual well-being and quality of life; because symptom burden may also lie on the pathway from spiritual well-being to quality of life, Model 2 is reported as a sensitivity analysis rather than as the primary estimate. Model 3 adds HADS-anxiety and HADS-depression, treated as putative intermediates; its coefficient for spiritual well-being is therefore a direct (mediator-adjusted) association rather than a total association and is expected to be attenuated. An extended Model 3 additionally includes residence and time since diagnosis. The assumed structure can be summarised as follows: sociodemographic and clinical covariates are treated as common causes of spiritual well-being, distress and quality of life; symptom burden as an antecedent of spiritual well-being, distress and quality of life; and anxiety and depression as intermediate between spiritual well-being and quality of life, with the explicit acknowledgement that the cross-sectional design cannot distinguish this ordering from its reverse. The tertile contrast and the inverse-probability-weighted contrast are descriptive comparisons on a different scale and are not the primary estimand. Multicollinearity was quantified with variance inflation factors, with a threshold of 5 taken as cause for concern. Predictor contributions to the explained variance of the primary outcome were partitioned by exact Lindeman–Merenda–Gold decomposition, averaging each predictor’s incremental R^2^ over all possible orderings of entry. Effect modification was tested by fitting multiplicative interaction terms and reporting the interaction *p* value together with subgroup-specific estimates; simple slopes were evaluated by re-centring the moderator at the 10th, 50th and 90th centiles. The functional form of the exposure–outcome relationship was examined with restricted cubic splines using four knots placed at the 5th, 35th, 65th and 95th centiles, and departure from linearity was tested by a likelihood-ratio test against the linear model.

Indirect associations were estimated with the product-of-coefficients method, understood here as a statistical decomposition of the cross-sectional association rather than as a counterfactual estimate of natural direct and indirect effects, because the identification assumptions of the latter (temporal precedence and the absence of unmeasured exposure–mediator, mediator–outcome and exposure–outcome confounding, including confounding induced by the exposure) cannot be verified in this design [12]. The decomposition is also non-discriminating: a model in which the roles of exposure and mediator are exchanged, in which distress and spiritual well-being are reciprocally related, or in which both share a common cause would generate the same pattern of covariances, so a significant indirect association indicates that the data are compatible with the hypothesised ordering, not that it is preferred over these alternatives. For each hypothesised pathway, the exposure–mediator (a), mediator–outcome (b), direct (c′) and total (c) effects were estimated from covariate-adjusted linear models, the indirect effect was computed as the product a × b, and its 95% confidence interval and two-sided *p* value were obtained from 5000 non-parametric bootstrap resamples using the percentile method. The proportion mediated is reported as the ratio of the indirect to the total effect. Because HADS-anxiety and HADS-depression are correlated (ρ = 0.60), the single-mediator models estimate overlapping indirect associations, and their proportions mediated are not independent and must not be summed. A parallel multiple-mediator model, in which both HADS subscales were entered simultaneously in the outcome model so that their specific indirect associations are mutually adjusted, was therefore added during peer review as a post hoc exploratory analysis and is reported alongside the single-mediator models. A further post hoc sensitivity analysis, also added in response to peer review, re-estimated the primary association and the anxiety pathway with the peace subscale removed from the exposure (the eight-item meaning-plus-faith score, standardised within the sample), because the peace items share affective content with the HADS and the emotional components of quality of life; the extent to which the estimates shrink when peace is excluded gives a rough indication of how much of the observed pathway may reflect shared item content. Bootstrapping was preferred to the Sobel test because the sampling distribution of a product of coefficients is asymmetric in samples of this size. Partial correlations between spirituality domains and outcome domains were computed as Pearson correlations between residuals from covariate-adjusted models, with degrees of freedom reduced by the number of covariates.

Missing data were minimal: 7 participants lacked a valid date of diagnosis and one lacked a role functioning score, giving a maximum of 4.0% missingness for any analysis variable. The primary analyses were therefore complete-case. Because none of the variables in Models 1 and 2 had missing values, multiple imputation cannot alter those models; the multiple-imputation sensitivity analysis was instead applied to the extended Model 2 (Model 2 covariates plus residence and time since diagnosis), the specification in which missingness actually arises, and its complete-case counterpart (*n* = 168) is reported alongside it. Time since diagnosis (*n* = 7) and role functioning (*n* = 1) were imputed by chained equations (predictive mean matching) in 20 imputed datasets, with an imputation model containing all analysis variables including the outcome; estimates were pooled by Rubin’s rules. Eight further sensitivity analyses were prespecified: model-based rather than robust standard errors; exclusion of patients in remission; restriction to ECOG 0–2; additional adjustment for tumour site and education; additional adjustment for anxiety and depression; median quantile regression; a non-parametric bootstrap with 2000 resamples; and an inverse-probability-of-treatment-weighted contrast of the upper versus the lower half of the FACIT-Sp-12 distribution. The propensity model was a logistic regression of upper-half membership on age, sex, advanced disease, ECOG performance status, comorbidity count, residence and time since diagnosis; because time since diagnosis was missing for seven participants, the propensity model, the weights and the weighted contrast were estimated separately within each of the 20 imputed datasets and pooled by Rubin’s rules, so that all 175 participants contributed, and a complete-case weighted analysis (*n* = 168) was performed as a check; stabilised weights were truncated at the 1st and 99th centiles; positivity was checked by inspecting the overlap of the propensity-score distributions; covariate balance was assessed by standardised mean differences before and after weighting, with a threshold of 0.10; the effective sample size was computed by Kish’s formula; and the variance of the weighted contrast was estimated with a robust sandwich estimator. Weight diagnostics, averaged across the imputed datasets, are reported alongside the sensitivity analyses, and the analytic sample, model covariates and missing-data handling of every sensitivity analysis are listed in Section 3.9. All tests were two-sided with α set at 0.05. The confirmatory analyses were the four prespecified tests specified in the Introduction: the primary association (H1, Model 1), the two single-mediator indirect associations through HADS-anxiety (H2a) and HADS-depression (H2b), and the spiritual well-being × symptom burden interaction (H3). The primary test was performed at α = 0.05 without adjustment. The three secondary confirmatory tests address distinct scientific questions and were each prespecified as a single test, so their unadjusted *p* values are reported as the primary inference; because they nonetheless form a small family, we also report whether their conclusions change under Holm–Bonferroni adjustment across the three tests (they do not: adjusted *p* < 0.001 for H2a and H2b and *p* = 0.649 for H3). All remaining analyses are exploratory, whether prespecified or introduced during peer review, and the latter are identified as post hoc where reported. For these, false-discovery-rate control by the Benjamini–Hochberg procedure [22] was applied within each family of tests (the eight subgroup interaction tests, the 36 subscale-by-outcome models and the 25 between-tertile comparisons), and results are interpreted with reference to the adjusted q values and to the magnitude and precision of the estimates rather than to unadjusted threshold significance. Exact *p* values are reported throughout. Analyses were performed in Python (version 3.11) using the pandas (version 2.2.2), statsmodels (version 0.14.2) and SciPy (version 1.13.1) libraries.

## 3. Results

For orientation, the confirmatory results of this study are confined to three places: the primary Model 1 estimate (H1; first paragraph of Section 3.4), the two single-mediator indirect associations through anxiety and depression (H2a and H2b; Section 3.8) and the spiritual well-being × symptom burden interaction (H3; Section 3.9). Everything else in this section—the tertile comparisons, subgroup analyses, subscale-specific models, correlation matrix, variance decomposition, remaining mediation pathways, spline analysis and sensitivity analyses—is exploratory or supportive and is reported to document robustness, not to add to the confirmatory inference.

### 3.1. Cohort Characteristics

Between recruitment and database lock, 175 patients completed the assessment battery and were included; item-level data were essentially complete, with a valid date of diagnosis missing for seven patients and a role functioning score for one. Their demographic and clinical characteristics, presented overall and across the three spiritual well-being tertiles, are summarised in Table 1.

The cohort had a mean age of 62.2 ± 13.1 years; 114 patients (65.1%) had metastatic or terminal disease, and 90 (51.4%) screened positive for clinically significant psychological distress, defined as a score of eight or more on either HADS subscale (HADS-anxiety ≥ 8 in 55 patients, 31.4%; HADS-depression ≥ 8 in 78, 44.6%; both in 43, 24.6%). Across spiritual well-being tertiles, age, sex, residence, education, tumour site, disease status, comorbidity burden and treatment modality were similar. Significant between-group differences were observed for ECOG performance status (*p* = 0.006), service attendance (*p* = 0.006), seeking spiritual support (*p* < 0.001) and change in religious practice (*p* = 0.011) (Table 1). For the variables with sparse cells (marital status, primary tumour site, disease status and religious affiliation), exact tests gave the same inference as Pearson’s χ^2^ (all *p* > 0.05).

### 3.2. Patient-Reported Outcomes Across Spiritual Well-Being Tertiles

The distribution of every EORTC QLQ-C30 scale, the HADS subscales and the remaining spirituality measures across the three exposure groups is reported in Table 2.

Global health status/quality of life increased from 53.8 ± 28.0 in the low spiritual well-being tertile to 74.5 ± 23.1 in the high tertile (*p* < 0.001). The functional summary score increased from 52.5 ± 22.4 to 75.3 ± 16.6, while composite symptom burden decreased from 38.1 ± 22.9 to 18.4 ± 16.0 (both *p* < 0.001). HADS-anxiety decreased from 9.0 ± 5.5 to 2.9 ± 2.7 and HADS-depression from 10.9 ± 4.6 to 6.2 ± 4.3 (both *p* < 0.001) (Table 2).

### 3.3. Primary and Secondary Outcome Comparisons

Formal unadjusted and covariate-adjusted comparisons of the primary and secondary outcomes between the high and low spiritual well-being groups, with standardised effect sizes, are presented in Table 3.

These tertile contrasts are descriptive; the primary estimand is the continuous-exposure coefficient reported in Section 3.4. After covariate adjustment, the high versus low spiritual well-being tertile differed by 14.9 points in global quality of life (95% CI 4.3 to 25.5; *p* = 0.006). Adjusted differences were 16.5 points for the functional summary score, −16.2 points for composite symptom burden and 25.6 points for emotional functioning (all *p* < 0.001). HADS-anxiety and HADS-depression were lower by 5.7 and 4.1 points, respectively (both *p* < 0.001) (Table 3).

### 3.4. Primary Analysis and Exploratory Subgroup Analyses

In the prespecified primary model (Model 1, adjusted for age, sex, advanced disease, ECOG performance status and comorbidity count), each standard deviation increase in FACIT-Sp-12 was associated with 7.15 additional points of global quality of life (95% CI 3.45 to 10.86; *p* < 0.001). This is the primary estimand of the study; it corresponds to the total association c in Section 3.8, and it is the estimate to which every secondary analysis reported below should be referred.

Prespecified exploratory subgroup analyses with formal interaction tests (Table 4) examined whether the association was consistent across clinically relevant strata; they were not powered to detect effect modification, and no stratum-specific estimate should be read as evidence that the association is confined to, or absent from, any group.

The subgroup models in Table 4 omitted comorbidity count to preserve degrees of freedom within strata, and the corresponding full-cohort estimate (7.18; 95% CI 2.52 to 11.85; *p* = 0.003) is virtually identical. Interpretation of the subgroup analyses rests on the interaction tests, not on whether the estimate reached significance within a given stratum. None of the eight interaction tests remained significant after false-discovery-rate adjustment. The unadjusted interaction *p* value for residence was 0.027 (q = 0.22), with estimates of 9.53 points in urban and 2.95 points in rural patients; this is one of eight uncorrected comparisons, the stratum-specific confidence intervals overlap widely, and it is reported solely as an exploratory, hypothesis-generating observation rather than as an effect modifier (Table 4).

### 3.5. Domain-Specific Associations of Spirituality Dimensions

Because the total FACIT-Sp-12 score conflates existential and religious content, the meaning, peace and faith subscales and the RCI-10 were entered separately against every functional, symptom and distress outcome; the resulting adjusted coefficients are given in Table 5, and the corresponding partial correlation structure is displayed in Figure 1.

Peace showed the largest adjusted coefficients for global quality of life (β = 7.73; *p* < 0.001), emotional functioning (β = 15.00; *p* < 0.001) and lower symptom burden (β = −7.04; *p* < 0.001). Meaning was also associated with global quality of life (β = 5.90; *p* = 0.020) and several functional and distress outcomes. Faith was associated mainly with lower anxiety and depression, while RCI-10 reached nominal significance for global quality of life and anxiety (Table 5). After Benjamini–Hochberg adjustment across the 36 models in Table 5, all associations with unadjusted *p* ≤ 0.021 remained significant (q < 0.05), whereas the two RCI-10 associations (*p* = 0.046 and 0.049) did not (q = 0.09). Because each cell in Table 5 comes from a separate model and the subscales are correlated (Table 6), differences between coefficients cannot be inferred from differences in their *p* values. In a post hoc mutually adjusted model of global quality of life (added during peer review) containing meaning, peace and faith simultaneously with the Model 1 covariates, the coefficients per SD were 2.87 (95% CI −3.31 to 9.05) for meaning, 5.96 (0.79 to 11.13) for peace and 0.42 (−4.06 to 4.90) for faith; a Wald test of the peace–faith difference gave *p* = 0.089 and of the peace–meaning difference *p* = 0.47. The larger coefficient for peace therefore describes observed magnitudes and is not established as a statistically significant difference between subscales.

In Figure 1, the strongest adjusted partial correlation was between peace and anxiety (r = −0.648), followed by peace and emotional functioning (r = 0.519), peace and depression (r = −0.445) and peace and cognitive functioning (r = 0.437). Associations for faith and RCI-10 were smaller across most outcome domains.

### 3.6. Correlation Structure

The zero-order rank correlation structure among the spirituality measures, quality of life domains, distress measures and performance status is presented in Table 6.

FACIT-Sp-12 total correlated with meaning, peace and faith at ρ = 0.79, 0.82 and 0.78, respectively. Peace had the strongest correlation with anxiety (ρ = −0.67). Global quality of life correlated with FACIT-Sp-12 total at ρ = 0.38 and with meaning and peace at ρ = 0.40 each. Symptom burden correlated with global quality of life at ρ = −0.59 and with the functional summary score at ρ = −0.72 (Table 6).

### 3.7. Multivariable Model of Global Quality of Life

An extended Model 3 containing spiritual well-being, symptom burden, both distress subscales and all sociodemographic and clinical covariates, including residence and time since diagnosis, was fitted to the primary outcome (Table 7). The partition of explained variance among predictors is displayed in Figure 2. Because the Lindeman–Merenda–Gold decomposition requires complete cases and the two additional covariates contributed less than one percentage point of explained variance, the decomposition was performed on the nine-predictor Model 3 without residence and time since diagnosis (*n* = 175; R^2^ = 0.422); the two models are therefore not identical, and Figure 2 should be read as a decomposition of Model 3 rather than of the extended model in Table 7.

The full multivariable model explained 43.1% of the variance in global quality of life (adjusted R^2^ = 0.39). Composite symptom burden (β = −7.85 per SD; *p* = 0.012) and ECOG performance status (β = −4.86 per category; *p* = 0.014) remained significant. The spiritual well-being coefficient was 1.17 points per SD (95% CI −4.10 to 6.44; *p* = 0.664) after inclusion of both HADS subscales (Table 7). As expected for a mediator-adjusted (direct) association, this coefficient is not comparable with the primary estimand, and its attenuation is the anticipated consequence of conditioning on variables that lie on the hypothesised pathway.

The Lindeman–Merenda–Gold decomposition in Figure 2 attributed 12.98 percentage points of outcome variance to composite symptom burden and 8.88 points to ECOG performance status. HADS-anxiety and HADS-depression contributed 7.71 and 6.61 points, respectively. Spiritual well-being contributed 4.34 percentage points, corresponding to 10.3% of the total explained variance.

### 3.8. Mediation Analysis

Five prespecified single-mediator pathways—the two pathways from spiritual well-being through HADS-anxiety and HADS-depression to global quality of life constituting the confirmatory tests H2a and H2b, and the remaining three being prespecified exploratory pathways—and one post hoc parallel-mediator model added during peer review were estimated with bootstrapped confidence intervals to examine whether emotional distress statistically accounts for the cross-sectional association between spiritual well-being and global quality of life (Table 8).

In the two confirmatory single-mediator models, the indirect association of spiritual well-being with global quality of life was 4.21 points (95% CI 1.89 to 6.86; *p* < 0.001) through HADS-anxiety and 3.03 points (95% CI 1.17 to 5.43; *p* < 0.001) through HADS-depression, against a total association of 7.15 points; the corresponding proportions in Table 8 are descriptive ratios, come from separate models with correlated mediators, and overlap. In the post hoc parallel-mediator model the joint indirect association through both subscales was 4.46 points (95% CI 2.10 to 7.02; *p* < 0.001), leaving a direct association of 2.69 points (−1.42 to 6.80); the remaining exploratory pathways are given in Table 8 without further comment. All indirect associations are cross-sectional statistical decompositions: they show that the data are compatible with the hypothesised ordering, but they are equally consistent with the reverse ordering, with reciprocal relationships between distress and spiritual well-being, and with common causes of both (Section 4.1), and they do not establish temporal ordering.

In the post hoc sensitivity analysis addressing the affective content shared by the peace items and the HADS, the exposure was re-defined as the eight-item meaning-plus-faith score with peace excluded. Its Model 1 association with global quality of life was 5.12 points per SD (95% CI 1.31 to 8.93; *p* = 0.009), and the bootstrapped indirect association through HADS-anxiety was 2.41 points (95% CI 0.78 to 4.35; *p* = 0.003), corresponding to 47.1% of the total association compared with 58.9% for the full FACIT-Sp-12. The association therefore persisted, with a smaller total coefficient and a smaller indirect component, when the subscale with the greatest affective overlap was removed.

### 3.9. Dose–Response, Moderation and Sensitivity Analyses

The analyses in this section are secondary and are reported in condensed form; none alters the inference from the primary model. The robustness of the symptom-burden-adjusted estimate (Model 2) was interrogated through the reference model and nine prespecified sensitivity analyses (Table 9 and Table 10), the functional form of the exposure–outcome relationship was examined with restricted cubic splines (Figure 3), and the competing buffering hypothesis was tested directly (Figure 4).

The Model 2 reference estimate, adjusted for symptom burden in addition to the Model 1 covariates, was 4.67 points of global quality of life per SD of spiritual well-being (95% CI 0.37 to 8.96; *p* = 0.033). The other sensitivity estimates on the per-SD scale ranged from 4.55 to 6.64 points, with two exceptions expected on design grounds: attenuation in the ECOG 0–2 restriction, which truncates the range of both exposure and outcome, and after adjustment for anxiety and depression, as expected when conditioning on putative intermediates (Table 9). The multiple-imputation estimate (4.55) differs from the Model 2 reference (4.67) only because it was fitted to the extended Model 2; its complete-case counterpart was 4.49 (Table 10).

Figure 3 shows an increasing adjusted dose–response across the observed FACIT-Sp-12 range. Relative to the cohort median, the estimated difference was −9.0 points at the 10th centile and +11.5 points at the 90th centile. The test for non-linearity was not significant (χ^2^ = 1.35; *p* = 0.510). This indicates no evidence of departure from linearity rather than confirmation that the relationship is linear; precision is limited at the lower end of the score distribution, where few patients were observed, and the ceiling effect at the upper end restricts the range over which curvature could be detected.

In Figure 4, no statistically detectable spiritual well-being × symptom burden interaction was found in this sample (β = −1.198, 95% CI −6.355 to 3.959; *p* = 0.649). Because higher symptom-burden scores denote worse symptoms and higher global scores denote better quality of life, the symptom-burden slope is negative, and buffering would correspond to a positive interaction coefficient (a slope that becomes less steep as spiritual well-being increases). The confidence interval therefore spans values from an exacerbating (slope-steepening) interaction of about 6 points to a buffering interaction of about 4 points per SD × SD, so clinically meaningful effect modification in either direction cannot be excluded. The point estimate lay, if anything, in the direction opposite to buffering, but it was small and imprecise. The estimated symptom-burden slopes were −8.0, −9.9 and −11.1 quality-of-life points per SD at the 10th, 50th and 90th centiles of spiritual well-being, respectively. The slight steepening across centiles is consistent with the sign of the interaction coefficient.

## 4. Discussion

### 4.1. Analysis of Findings

The principal finding is the primary Model 1 estimate: each standard deviation of spiritual well-being was associated with 7.15 points (95% CI 3.45 to 10.86) of global quality of life after adjustment for baseline confounders, an association that the secondary and sensitivity analyses reproduced in direction and, with the expected exceptions, in magnitude. The two prespecified secondary hypotheses yielded two findings that we report separately. First, anxiety and depression statistically accounted for substantial, overlapping proportions of the spiritual well-being–quality-of-life association and, consistently, the direct coefficient for spiritual well-being was markedly attenuated once anxiety and depression were entered into the fully adjusted model. Second, no statistically detectable multiplicative interaction between spiritual well-being and symptom burden was found, with a confidence interval wide enough to include both buffering and its opposite. These two findings derive from different tests with very different precision, and a significant indirect association alongside a non-significant interaction does not establish that one explanation is better supported than the other: the indirect associations are compatible with, but do not demonstrate, the mediation account, and the interaction analysis is inconclusive with respect to the buffering account. Because all variables were measured at one time point, these findings should be interpreted as associations compatible with the proposed model rather than as a demonstrated mechanism; the reverse ordering, in which lower distress or better quality of life makes it easier to experience meaning and inner peace, is equally consistent with the data.

This pattern is consistent with prior oncology research in which existential aspects of spirituality are closely linked to psychological adjustment. Jimenez-Fonseca and colleagues reported mediation involving spiritual well-being and mental adjustment [23], Scheffold and colleagues described spiritual well-being as an intermediate factor between attachment insecurity and distress in advanced cancer [24], and path-analytic work has likewise identified indirect routes linking spiritual well-being to quality of life [25]. The close association with anxiety and depression in our cohort also parallels observations in end-of-life cancer populations [26] and multicentre data relating spiritual pain to symptom distress and quality of life [27]. Taken together, these studies and our results suggest that emotional appraisal, worry, hopelessness and loss of inner coherence may be important correlates through which existential well-being and perceived quality of life travel together.

Longitudinal evidence provides a useful frame for temporal plausibility. In a prospective cohort of women with breast cancer beginning chemotherapy in the United States, faith at baseline was associated with meaning and peace three months later and, indirectly through them, with mental quality of life at 15 months [28]. That ordering—faith preceding meaning and peace, which precede later quality of life—is compatible with the cross-sectional pattern observed here. It does not, however, resolve the causal limitations of the present design, and its sample (early-stage breast cancer, a religiously plural population, a mental-component quality-of-life outcome) differs substantially from ours. Reverse causality remains a live possibility: patients with less emotional distress or better quality of life may be more able to find meaning in the illness experience or to feel at peace, so that the direction of association assumed in our mediation models could run wholly or partly the other way.

It is worth setting out explicitly the alternative causal structures with which the mediation results are equally compatible, because the product-of-coefficients decomposition cannot discriminate among them [12]. At least four structures would reproduce the observed pattern of associations. In the first, the hypothesised one, spiritual well-being lowers anxiety and depression, which in turn worsen quality of life. In the second, the ordering is reversed: anxious and depressive symptoms erode the capacity to feel at peace or to find meaning, and poor quality of life does the same, so that spiritual well-being is a consequence rather than a cause of distress. In the third, spiritual well-being and distress are reciprocally related over time, each shaping the other in a feedback loop that a single measurement occasion collapses into one cross-sectional correlation. In the fourth, a common antecedent—physical symptom burden, functional decline, personality traits such as neuroticism or optimism, social support, or simply the negative-affect content shared by the questionnaires—drives spiritual well-being, distress and quality of life together, so that the “indirect” association is a by-product of confounding rather than a pathway; the symptom-burden decompositions in Table 8, in which symptom burden was associated with spiritual well-being, distress and quality of life alike, illustrate this possibility directly. Under each of these alternatives the indirect coefficients in Table 8 would remain statistically significant and of similar magnitude, which is why we present them as descriptive decompositions rather than as evidence for one ordering over another. Cross-lagged panel or intensive longitudinal designs, in which spiritual well-being and distress are measured repeatedly and each is modelled as a predictor of subsequent change in the other, would be required to distinguish among these structures.

The domain-specific analyses further refine this interpretation. Peace showed the largest coefficients for global quality of life, emotional functioning, anxiety and depression, and meaning a similar but smaller pattern; because the subscales were examined in separate models and are substantially correlated, and because the mutually adjusted comparison of peace with faith did not reach significance (Section 3.5), these differences describe observed magnitudes rather than established differences between subscales. Faith and formal religious commitment were substantially less informative for most functional and symptom outcomes. This distinction is compatible with previous FACIT-Sp research showing that peace and meaning are more strongly related to quality of life and coping than faith [29], with survivorship analyses in which faith appears to operate indirectly through meaning [30], and with work supporting the clinical relevance of the existential dimensions of the scale [31]. The finding is particularly relevant in this Romanian cohort, where religious affiliation was highly prevalent. In such a setting, formal religious identity may vary less than the subjective experience of peace, meaning or spiritual struggle. Religious commitment should therefore not be assumed to be interchangeable with spiritual well-being, and high religious involvement does not necessarily indicate absence of existential distress. This is also consistent with evidence that negative religious coping can accompany poorer psychological outcomes [32] and that demoralisation can remain clinically important independently of religious belief [33].

A further interpretive consideration arises from the confessional composition of the cohort. Orthodox theology has a distinctive account of suffering, in which affliction may be understood not merely as an evil to be avoided but as a potentially meaning-bearing experience; a patient who holds a “why” of this kind may bear the “how” of illness differently from one who does not. If so, the meaning ascribed to suffering could shape how meaning and peace relate to distress and quality of life, and the inductive reach of our findings would be bounded by the Orthodox majority of the sample. We could not test this adequately: only 33 participants (18.9%) were non-Orthodox, and a post hoc interaction (added during peer review) between Orthodox affiliation and spiritual well-being added to the Model 1 regression was small and imprecise (7.6 points per SD in Orthodox and 5.9 in non-Orthodox participants; *p* for interaction = 0.71), which is uninformative at the power available. Denominational affiliation is, moreover, a coarse proxy for the theological understanding of suffering that an individual actually holds. Studies that measure meaning-in-suffering directly, and that recruit comparable numbers of Orthodox and non-Orthodox patients, would be required to examine this question.

A related conceptual question is whether constructs entered into the same models need to be of the same psychological kind. Peace, as operationalised in the FACIT-Sp, is largely an affective state—closer in kind to well-being, or to the absence of inner disturbance—whereas meaning has a more cognitive and discursive character, a judgement about purpose and coherence; the three-factor work on the scale characterised them in these terms [17]. Our regression and mediation methods do not require predictors or mediators to be alike in kind: each is treated as a continuous score whose linear association with the outcome is estimated separately, and heterogeneity of kind does not bias those estimates. Kind matters in two other places, however. First, the FACIT-Sp-12 total score aggregates affective, cognitive and religious content into a single number, so its association with quality of life is a weighted average of components that may relate to outcomes for different reasons; this is why the subscale analyses are reported. Second, the closer a predictor is in kind to the outcome, the more its association may reflect shared content rather than a substantive relationship—an issue that bears most directly on peace and to which we return under construct overlap in the limitations. What the methodology cannot do is say whether the apophatic character of peace and the discursive character of meaning place them on a common metric of value; it can only describe how each covaries with the outcomes measured.

The consequences of this overlap for the present estimates deserve specific comment. The four peace items (feeling peaceful, having trouble feeling peace of mind, being able to reach deep down into oneself for comfort, and a sense of harmony within oneself) describe an affective state whose absence is close to what the HADS anxiety items measure (feeling tense, unable to relax, restless or beset by worrying thoughts), so the two scales overlap in content as well as in construct. Three effects can be anticipated. First, shared item content inflates the exposure–mediator path (a) for peace and, to a lesser degree, for the FACIT-Sp-12 total score, of which peace contributes a third: the observed peace–anxiety correlation (ρ = −0.67) is close to the ceiling set by the reliabilities of the two scales (α = 0.765 and 0.859), and correcting it for attenuation gives a value of approximately −0.83, implying that the two scales share roughly two-thirds of their reliable variance and have limited discriminant validity in this sample. Second, because the HADS items and the emotional functioning items also share content with the global health status/quality of life rating, the mediator–outcome path (b) is subject to the same inflation, so the indirect coefficients in Table 8, and particularly the peace–anxiety pathway, are better regarded as upper bounds on any substantive pathway than as unbiased estimates of it. Third, overlap acts in the same direction as the reverse-ordering and common-cause alternatives discussed above and cannot be separated from them without measures that are free of shared content. The post hoc analysis with peace removed from the exposure (Section 3.8) gives some purchase on the size of the problem: the association of the meaning-plus-faith score with global quality of life was smaller but persisted (5.12 points per SD; 95% CI 1.31 to 8.93), and the indirect association through anxiety fell from 58.9% to 47.1% of the total, which is consistent with a part, but not the whole, of the observed pathway being attributable to shared affective content. Approaches for handling spirituality measures contaminated with indicators of mental health have been proposed, including restricting the exposure to religious or existential content [34]; the faith and RCI-10 results, which are free of affective item content and were substantially weaker, indicate the lower bound that such an approach would yield here.

The absence of a statistically detectable spiritual well-being × symptom burden interaction is consistent with, but does not prove, an additive relationship; the confidence interval was wide and the interaction test underpowered, so buffering cannot be excluded. The point can be made quantitatively. From the standard error of the interaction term (approximately 2.6 points), the smallest interaction detectable with 80% power at α = 0.05 was about 7.4 points per SD × SD, whereas an interaction of 3 to 4 points per SD × SD, which would flatten the median symptom-burden slope (−9.9 points per SD) by roughly a third for each SD of spiritual well-being and would be clinically meaningful, lies within the reported confidence interval. The non-significant interaction is therefore an absence of evidence, not evidence against buffering. Independently of the statistical outcome, spiritual care is a complementary component of multidimensional supportive care and not a substitute for symptom control. In the present data, higher spiritual well-being was associated with an upward shift in predicted quality of life, but the decline in quality of life accompanying greater physical symptom burden was similar across low, median and high levels of spiritual well-being. The two domains appeared additive within the precision of this sample, but that precision was low, and the buffering account is neither supported nor rejected by these data; the two explanatory accounts remain open and should be regarded as competing on equal terms. Either account is compatible with a model of supportive oncology in which symptom management, psychological assessment and existential or spiritual care are delivered in parallel. These data support routine assessment of spiritual and emotional concerns; they do not show that interventions which increase spiritual well-being will reduce anxiety or depression or improve quality of life, a question that only interventional studies can answer. Meaning-centred psychotherapy and dignity therapy provide examples of interventions that specifically address existential and emotional processes [35,36], while routine distress screening remains an important component of comprehensive oncology care [37]. The comparatively weaker associations between spirituality and physical symptom domains also accord with prior evidence in symptom-specific areas such as cancer-related fatigue [38].

The dose–response analysis adds a further practical point. There was no evidence of departure from linearity in the association between FACIT-Sp-12 and global quality of life across the observed range, and no statistically supported threshold or plateau, although the analysis had limited precision at the lower end and the ceiling effect restricted the upper end. This argues against imposing a single study-derived cut-point for identifying patients who may benefit from further assessment. A continuous score, supplemented by clinical discussion of meaning, peace, faith and spiritual concerns, may preserve more information than a simple high-versus-low classification. At the same time, the wider uncertainty at the lower end of the score distribution indicates that the precise shape of the association in patients with very low spiritual well-being remains less certain and should be examined in larger cohorts enriched for severe existential distress. Ceiling effects in the high tertile (Section 2.3) also mean that variation in meaning, peace and faith is compressed at the upper end of the scale; this restricts the range over which the regression and spline models can estimate slopes, attenuates associations at the upper end, reduces the sensitivity of the subgroup and subscale analyses, and makes the high tertile a less discriminating category than its label suggests.

The subgroup analyses were exploratory and should be read as such. None of the eight interaction tests remained significant after false-discovery-rate adjustment, and the stratum-specific confidence intervals were wide and overlapping, so the data provide no reliable evidence of effect modification by any characteristic examined. Residence was the only interaction with an unadjusted *p* value below 0.05 (q = 0.22). We note it only because territorial inequalities in access to oncology, palliative and psychosocial services have been documented in Romania [5,15,39] and rural cancer survivors may report poorer health-related quality of life [40], and because the rural subgroup was small (*n* = 75), residence was dichotomised, and no measures of distance to services, socioeconomic position or social support were collected. A statistically fragile result of this kind is a hypothesis for multicentre studies with direct measures of access, not a finding of this study, and we draw no clinical inference from it.

Several features strengthen the internal consistency of the findings. The primary association was examined on both categorical and continuous exposure scales, remained present after adjustment for major clinical covariates and symptom burden, and was evaluated with robust standard errors, bootstrap procedures, multiple imputation, quantile regression and inverse-probability weighting. The agreement of these approaches reduces the likelihood that the principal result is driven by one modelling choice. At the same time, the attenuation observed after adjustment for anxiety and depression is not evidence that spiritual well-being becomes clinically irrelevant; rather, it identifies the strong overlap among existential well-being, emotional distress and patient-reported quality of life that should be examined prospectively. Future longitudinal studies should determine temporal ordering, identify which patients benefit most from targeted spiritual or existential interventions, and test whether changes in anxiety or depression mediate subsequent changes in quality of life.

With respect to originality, the indirect relationship linking faith, meaning, peace and quality of life has been documented before [28,29,30], and we do not claim to have discovered it. The contribution of this study lies elsewhere: in examining the buffering and mediation accounts in the same cohort with prespecified hypotheses, reporting each finding on its own terms rather than ranking the two accounts; in separating institutional religious commitment (RCI-10) from existential well-being (FACIT-Sp-12) in a setting where religious affiliation is close to a cultural constant, so that residual associations of meaning and peace cannot be attributed to religious belonging alone; and in extending the evidence base to a predominantly Orthodox, Eastern European population managed across oncology and palliative care services, which is largely absent from the literature. The scope of the conclusions is correspondingly bounded: they describe cross-sectional associations in this cultural, religious and sociodemographic context and do not speak to the universality of the pattern or to the efficacy of spiritual interventions.

### 4.2. Study Limitations

Several limitations qualify these findings. The design is cross-sectional, so although we use the language of mediation, all three variables in every pathway were measured simultaneously and the causal ordering is assumed rather than demonstrated; reverse pathways in which distress erodes spiritual well-being, or in which poor quality of life diminishes both, are statistically indistinguishable from the model we fitted and are clinically plausible. Longitudinal or, ideally, interventional data are required before any causal claim can be sustained. Recruitment took place at a single centre serving both oncology and palliative care in one Romanian city, and the cohort is culturally and religiously homogeneous, with more than four-fifths of participants Orthodox Christian; although religious commitment itself varied widely here (observed RCI-10 total range 10–46 of a possible 10–50), the cultural normativity of religious practice may limit how much its variation can explain in this setting, and the findings should not be extrapolated uncritically to religiously plural populations. The questionnaire was a locally adapted instrument battery, and this is the most consequential measurement limitation: the FACIT-Sp-12 was administered on a four-point rather than the validated five-point response format, so scores are reported in a 12–48 rather than a 0–48 metric and are not directly comparable with published norms; the RCI-10 total was recorded for the full 10-item instrument although only eight items were retained at item level, so internal consistency could be estimated for those eight items only; several EORTC QLQ-C30 items were administered on a non-standard response format and required rescaling; one physical functioning item was absent; and three HADS items had been recorded against an inverted response column and were re-keyed on psychometric grounds. The direction of bias introduced by these deviations cannot be assumed: non-standard response formats, a missing item and post hoc recoding can bias estimates toward or away from the null and can affect validity unpredictably, and the internal consistency observed in this sample does not establish the validity of the modified formats. The findings should accordingly be read as pertaining to locally adapted instruments rather than to the validated FACIT-Sp-12, QLQ-C30 and HADS: construct validity, and hence comparability with the published literature, has not been demonstrated for the adapted formats, and neither the absolute scores nor the effect sizes reported here can be assumed to be equivalent to those obtained with the standard versions. Replication with the validated formats is therefore a priority, and we regard it as a prerequisite before any of the present estimates are compared quantitatively with other cohorts. All exposures and outcomes are self-reported and were collected at a single sitting, so shared-method variance may inflate the correlations among them, and unmeasured confounders such as social support, financial strain, personality traits and pre-existing psychiatric history were not available. Construct overlap is a related concern: the FACIT-Sp peace items (feeling peaceful, a sense of harmony, being able to reach down deep for comfort) share affective content with the HADS anxiety and depression items and with the emotional and global components of patient-reported quality of life, so part of the strength of the observed associations, and particularly of the peace–anxiety correlation (ρ = −0.67), may reflect measurement of overlapping constructs at the same sitting rather than distinct phenomena [34]. The disattenuated peace–anxiety correlation of approximately −0.83 (Section 4.1) indicates that discriminant validity between these two scales is limited in this sample, so the mediation estimates involving peace, and to a lesser extent the total score, should be regarded as upper bounds; the post hoc analysis with peace excluded reduced but did not abolish the association, and the residual estimate is itself subject to the remaining sources of shared-method variance. The absence of measures of social support and optimism is a specific limitation. Both are associated with spiritual well-being in patients with cancer [41], and in a setting such as this one spirituality and religious commitment may index not only intrapersonal resources but also community belonging, social support and religious participation; the associations we report may therefore partly reflect these unmeasured psychosocial resources, and future studies should measure them directly. Selection bias is also possible: patients unable to complete the questionnaire, and those judged by their clinician to be too burdened to do so, were excluded, so that patients with the poorest performance status, greatest symptom burden or most severe existential distress are likely to be under-represented, which may have compressed the lower end of every distribution and attenuated or distorted the associations. The sample size was determined by feasibility rather than by a power calculation, which leaves the subgroup and interaction analyses—including the urban–rural finding—underpowered and vulnerable to both type I and type II error; the smallest detectable interaction (about 7.4 points per SD × SD; Section 4.1) exceeds several clinically plausible buffering effects, so the non-significant interaction must not be read as evidence against buffering, and conclusions about the relative merits of the two explanatory accounts are correspondingly provisional; false-discovery-rate control was applied to the exploratory analyses, whereas the four confirmatory tests (H1, H2a, H2b and H3) were interpreted at an unadjusted α of 0.05, although Holm–Bonferroni adjustment across the three secondary tests would not alter their inference (Section 2.4). Finally, external validation in independent, multicentre and culturally diverse cohorts is required before these estimates are used to guide practice.

## 5. Conclusions

In a Romanian cohort of patients with predominantly advanced cancer, higher scores on the adapted FACIT-Sp-12 were cross-sectionally associated with better global quality of life, better functioning and lower emotional distress. Anxiety and depression statistically accounted for part of this association, but temporal ordering and causality cannot be determined from these data, and the reverse ordering is equally compatible with them. No statistically significant interaction between spiritual well-being and symptom burden was identified, but the analysis had limited power and its confidence interval included clinically relevant buffering, so the buffering account is neither supported nor excluded and the two explanatory accounts remain open. Within the composite construct of spirituality, the observed coefficients were largest for inner peace and meaning and smallest for religious faith and formal religious commitment, a pattern that describes magnitudes rather than a formally established difference between subscales. These findings support assessing the existential dimensions of spiritual well-being separately from religious affiliation and attending to spiritual and emotional concerns alongside, not instead of, rigorous symptom control; whether interventions that increase spiritual well-being improve quality of life requires longitudinal and interventional study. Because the instruments were administered in locally adapted formats, these estimates pertain to the adapted measures and require confirmation with the validated versions before they are compared with other cohorts.

## Figures and Tables

**Figure 1:**
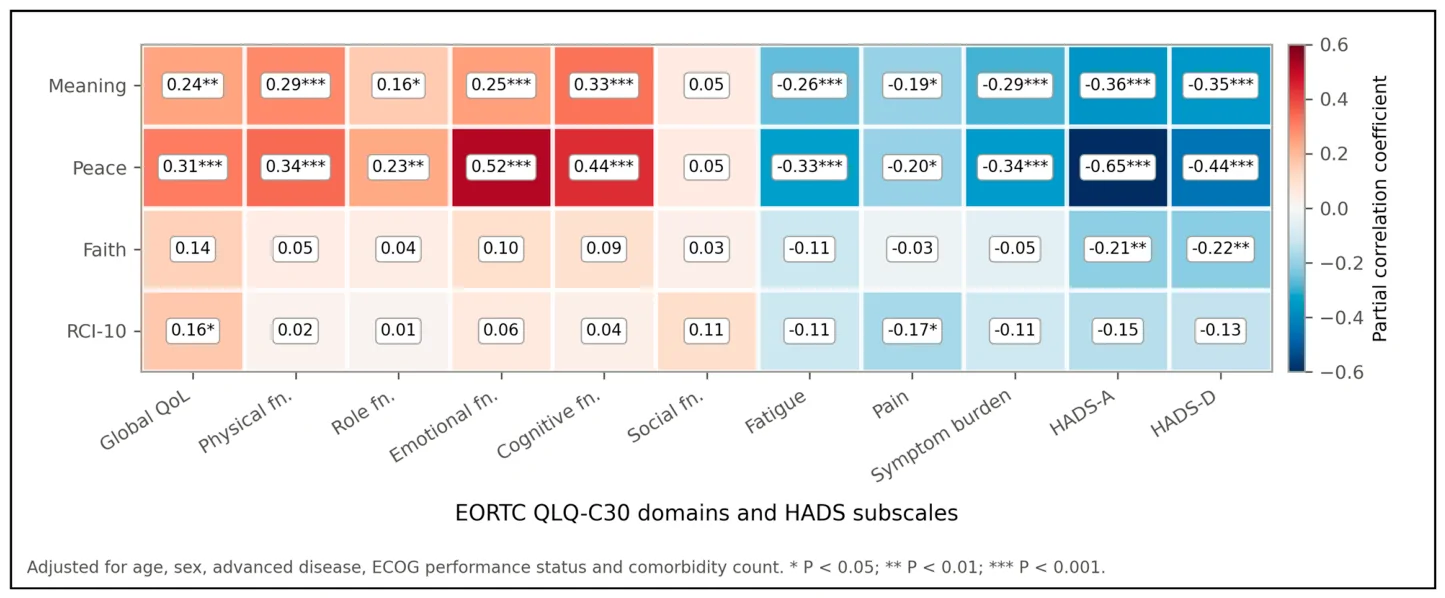
Partial correlations between the four spirituality dimensions and EORTC QLQ-C30 functional and symptom domains and HADS subscales, adjusted for age, sex, advanced disease, ECOG performance status and comorbidity count. Warm colours denote positive and cool colours negative partial correlations. * *p* < 0.05; ** *p* < 0.01; *** *p* < 0.001. Abbreviations: EORTC QLQ-C30, European Organisation for Research and Treatment of Cancer Quality of Life Questionnaire Core 30; HADS-A, Hospital Anxiety and Depression Scale—anxiety; HADS-D, Hospital Anxiety and Depression Scale—depression; RCI-10, Religious Commitment Inventory-10.

**Figure 2:**
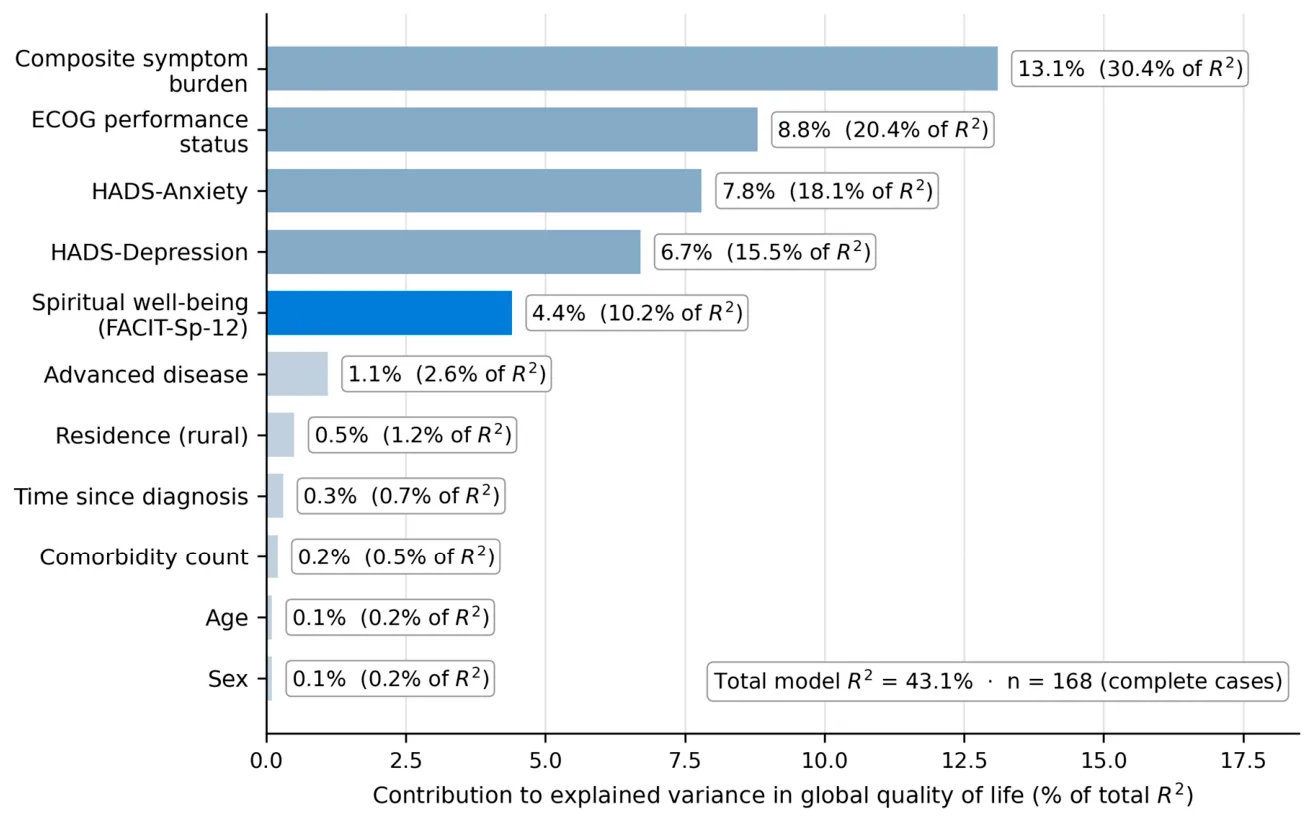
Exact Lindeman–Merenda–Gold decomposition of the variance in global health status/quality of life explained by each predictor, averaged over all possible orders of entry into the nine-predictor Model 3 (spiritual well-being, composite symptom burden, HADS-anxiety, HADS-depression, age, sex, advanced disease, ECOG performance status and comorbidity count; *n* = 175; R^2^ = 0.422), which differs from the extended model in Table 7 by the omission of residence and time since diagnosis. Bars show each predictor’s contribution in percentage points of outcome variance, with its share of the total explained variance in parentheses; the spiritual well-being contribution is highlighted. Abbreviations: FACIT-Sp-12, Functional Assessment of Chronic Illness Therapy—Spiritual Well-Being 12-item scale; HADS, Hospital Anxiety and Depression Scale.

**Figure 3:**
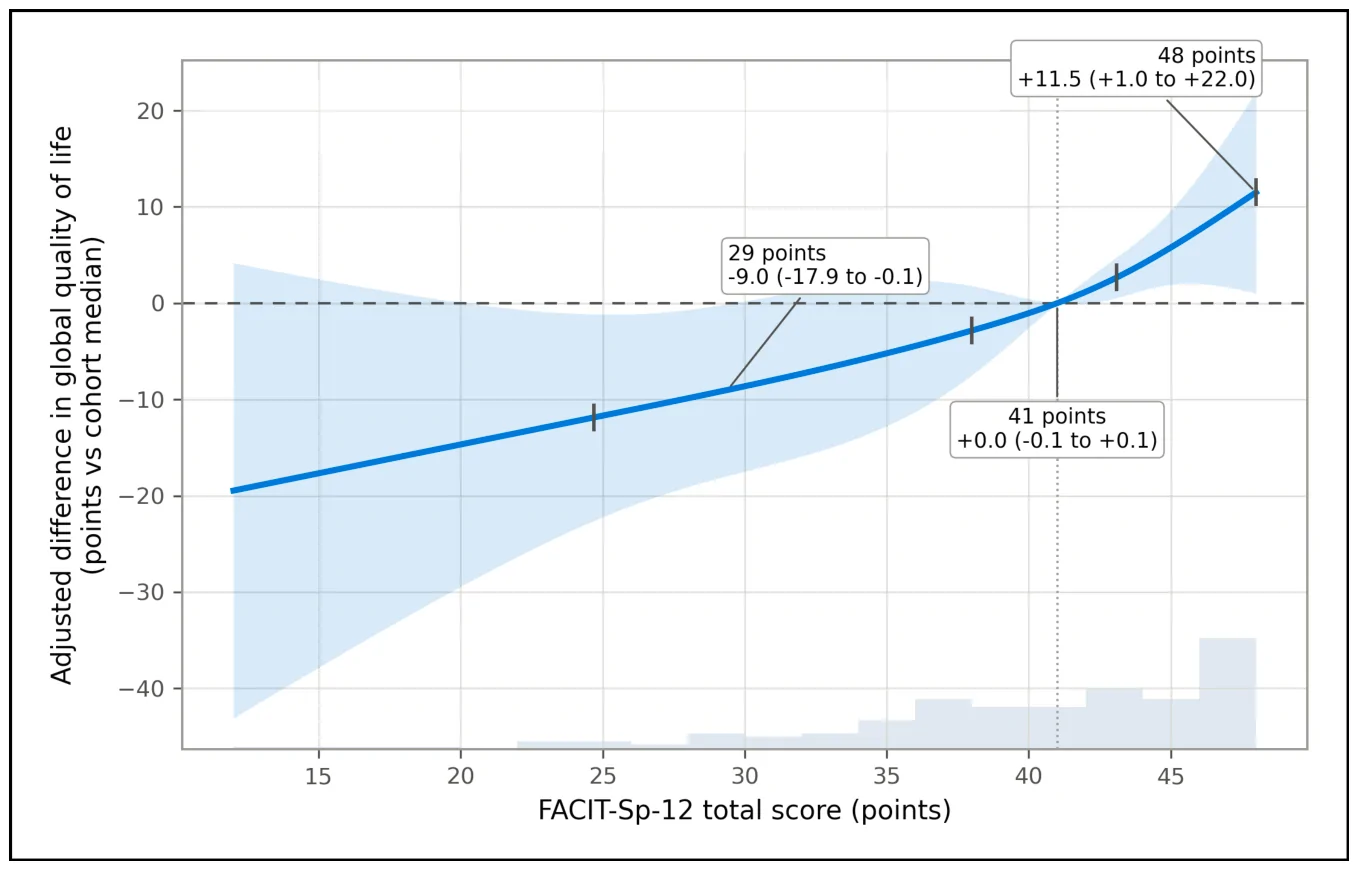
Adjusted dose–response relationship between FACIT-Sp-12 total score and global health status/quality of life, modelled with restricted cubic splines using four knots at the fifth, 35th, 65th and 95th centiles and referenced to the cohort median. The shaded band is the 95% confidence interval; vertical tick marks indicate knot positions and grey bars the sample distribution. Model adjusted for age, sex, advanced disease, ECOG performance status and comorbidity count. Abbreviations: FACIT-Sp-12, Functional Assessment of Chronic Illness Therapy—Spiritual Well-Being 12-item scale.

**Figure 4:**
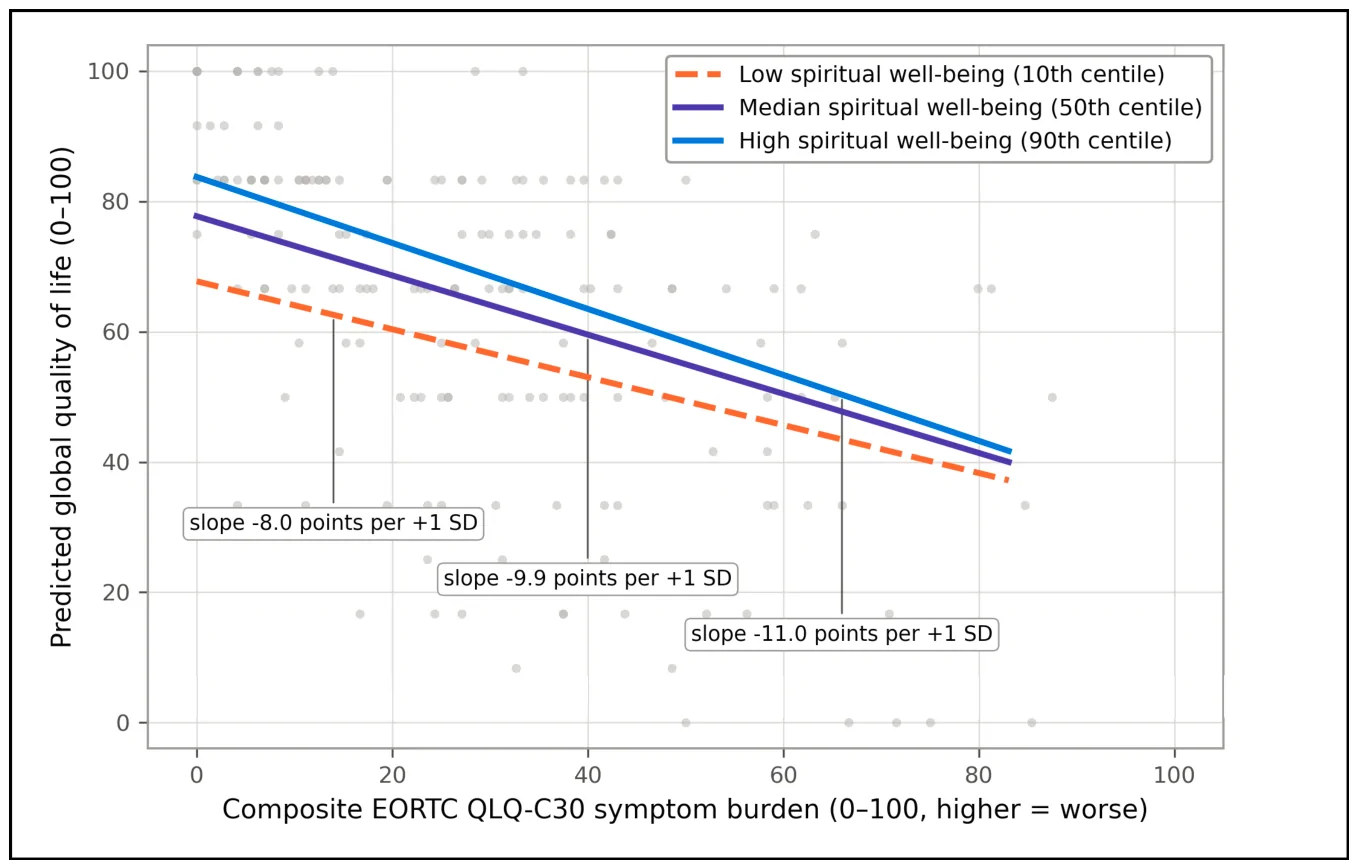
Test of the stress-buffering hypothesis: model-predicted global health status/quality of life as a function of composite symptom burden, evaluated at the 10th, 50th and 90th centiles of spiritual well-being. Grey points are individual observations. Near-parallel lines indicate the absence of a statistically detectable interaction in this sample; they do not establish strict additivity. Model adjusted for age, sex, advanced disease, ECOG performance status and comorbidity count with HC3 standard errors. Abbreviations: EORTC QLQ-C30, European Organisation for Research and Treatment of Cancer Quality of Life Questionnaire Core 30; SD, standard deviation.

**Table 1:** Demographic, clinical and religious characteristics of the study population, overall and by tertile of spiritual well-being.

Characteristic	Low SWB (*n* = 59)	Intermediate SWB (*n* = 66)	High SWB (*n* = 50)	Total (*n* = 175)	*p* Value	Effect Size
Age, years	62.1 ± 13.6	61.1 ± 12.6	63.7 ± 13.3	62.2 ± 13.1	0.579	0.006
Sex, *n* (%)						
Female	25 (42.4)	33 (50.0)	31 (62.0)	89 (50.9)	0.122	0.155
Male	34 (57.6)	33 (50.0)	19 (38.0)	86 (49.1)		
Body-mass index, kg/m^2^	23.8 ± 4.0	25.0 ± 4.3	25.6 ± 6.1	24.8 ± 4.8	0.127	0.024
Residence, *n* (%)						
Urban	36 (61.0)	37 (56.1)	27 (54.0)	100 (57.1)	0.743	0.058
Rural	23 (39.0)	29 (43.9)	23 (46.0)	75 (42.9)		
Education, *n* (%)						
Lower secondary	11 (18.6)	10 (15.2)	12 (24.0)	33 (18.9)	0.853	0.087
Vocational	14 (23.7)	14 (21.2)	8 (16.0)	36 (20.6)		
High school	24 (40.7)	27 (40.9)	19 (38.0)	70 (40.0)		
University	10 (16.9)	15 (22.7)	11 (22.0)	36 (20.6)		
Marital status, *n* (%)						
Married	31 (52.5)	40 (60.6)	33 (66.0)	104 (59.4)	0.086	0.178
Widowed	10 (16.9)	16 (24.2)	9 (18.0)	35 (20.0)		
Single	13 (22.0)	6 (9.1)	2 (4.0)	21 (12.0)		
Divorced	5 (8.5)	4 (6.1)	6 (12.0)	15 (8.6)		
Primary tumour site, *n* (%)						
Colorectal	14 (23.7)	18 (27.3)	12 (24.0)	44 (25.1)	0.882	0.137
Lung	10 (16.9)	10 (15.2)	10 (20.0)	30 (17.1)		
Breast	10 (16.9)	10 (15.2)	9 (18.0)	29 (16.6)		
Cervical/uterine	2 (3.4)	2 (3.0)	2 (4.0)	6 (3.4)		
Prostate	0 (0.0)	2 (3.0)	1 (2.0)	3 (1.7)		
Bladder	2 (3.4)	0 (0.0)	0 (0.0)	2 (1.1)		
Other	21 (35.6)	24 (36.4)	16 (32.0)	61 (34.9)		
Disease status, *n* (%)						
Localised	17 (28.8)	18 (27.3)	15 (30.0)	50 (28.6)	0.958	0.066
Metastatic	35 (59.3)	38 (57.6)	30 (60.0)	103 (58.9)		
Terminal	3 (5.1)	6 (9.1)	2 (4.0)	11 (6.3)		
Remission	4 (6.8)	4 (6.1)	3 (6.0)	11 (6.3)		
Time since diagnosis, months	28.0 (17.0–47.2)	23.5 (14.8–50.2)	28.5 (17.0–73.5)	25.0 (16.0–58.0)	0.309	0.041
ECOG performance status	2.0 (1.0–2.0)	1.0 (0.0–2.0)	1.0 (0.0–1.0)	1.0 (0.0–2.0)	0.006	0.062
Non-oncological comorbidities, *n*	2.0 (1.0–3.0)	2.0 (1.0–3.0)	2.0 (1.2–3.0)	2.0 (1.0–3.0)	0.562	0.006
Chemotherapy, *n* (%)						
Yes	43 (72.9)	52 (78.8)	35 (70.0)	130 (74.3)	0.537	0.084
No	16 (27.1)	14 (21.2)	15 (30.0)	45 (25.7)		
Surgery, *n* (%)						
Yes	24 (40.7)	20 (30.3)	14 (28.0)	58 (33.1)	0.309	0.116
No	35 (59.3)	46 (69.7)	36 (72.0)	117 (66.9)		
Radiotherapy, *n* (%)						
Yes	18 (30.5)	22 (33.3)	8 (16.0)	48 (27.4)	0.095	0.164
No	41 (69.5)	44 (66.7)	42 (84.0)	127 (72.6)		
Supportive care component, *n* (%)						
Yes	19 (32.2)	19 (28.8)	10 (20.0)	48 (27.4)	0.346	0.110
No	40 (67.8)	47 (71.2)	40 (80.0)	127 (72.6)		
Religious affiliation, *n* (%)						
Orthodox	50 (84.7)	55 (83.3)	37 (74.0)	142 (81.1)	0.117	0.171
Catholic	6 (10.2)	7 (10.6)	4 (8.0)	17 (9.7)		
Protestant/Neo-Protestant	3 (5.1)	4 (6.1)	6 (12.0)	13 (7.4)		
Other	0 (0.0)	0 (0.0)	3 (6.0)	3 (1.7)		
Service attendance, *n* (%)						
At least monthly	13 (22.0)	29 (43.9)	25 (50.0)	67 (38.3)	0.006	0.244
Less than monthly	46 (78.0)	37 (56.1)	25 (50.0)	108 (61.7)		
Sought spiritual support during illness, *n* (%)						
Yes	32 (54.2)	50 (75.8)	45 (90.0)	127 (72.6)	<0.001	0.320
No	27 (45.8)	16 (24.2)	5 (10.0)	48 (27.4)		
Change in religious practice, *n* (%)						
Intensified	24 (40.7)	29 (43.9)	35 (70.0)	88 (50.3)	0.011	0.217
New practices adopted	6 (10.2)	9 (13.6)	5 (10.0)	20 (11.4)		
Unchanged	22 (37.3)	26 (39.4)	9 (18.0)	57 (32.6)		
Diminished	7 (11.9)	2 (3.0)	1 (2.0)	10 (5.7)		

Data are mean ± standard deviation, median (interquartile range) or *n* (%) as indicated. *p* values are from one-way analysis of variance (continuous, symmetric), the Kruskal–Wallis test (ordinal or skewed) or Pearson’s χ^2^ test (categorical); for marital status, primary tumour site, disease status and religious affiliation, in which some cells had expected counts below five, the Fisher–Freeman–Halton exact test (Monte Carlo, 10,000 samples) gave the same inference (all *p* > 0.05). Effect sizes are η^2^ for analysis of variance, ε^2^ for Kruskal–Wallis tests and Cramér’s V for categorical variables. Abbreviations: ECOG, Eastern Cooperative Oncology Group; SWB, spiritual well-being (total score on the Functional Assessment of Chronic Illness Therapy—Spiritual Well-Being 12-item scale, FACIT-Sp-12).

**Table 2:** EORTC QLQ-C30 functional and symptom scales, psychological distress and spirituality measures by tertile of spiritual well-being.

Patient-Reported Outcome	Low SWB (*n* = 59)	Intermediate SWB (*n* = 66)	High SWB (*n* = 50)	Total (*n* = 175)	*p* Value	η^2^
Global health status/QoL	53.8 ± 28.0	65.9 ± 22.3	74.5 ± 23.1	64.3 ± 25.8	<0.001	0.102
Physical functioning	45.3 ± 29.6	62.2 ± 30.9	67.5 ± 25.2	58.0 ± 30.2	<0.001	0.095
Role functioning	34.2 ± 28.5	59.1 ± 33.4	62.7 ± 31.5	51.8 ± 33.5	<0.001	0.141
Emotional functioning	57.5 ± 31.9	72.9 ± 25.2	86.8 ± 16.5	71.7 ± 28.1	<0.001	0.171
Cognitive functioning	68.9 ± 27.4	87.1 ± 19.6	87.7 ± 16.4	81.1 ± 23.4	<0.001	0.140
Social functioning	55.6 ± 37.0	59.3 ± 37.2	72.0 ± 29.6	61.7 ± 35.6	0.044	0.036
Functional summary score	52.5 ± 22.4	68.1 ± 22.6	75.3 ± 16.6	64.9 ± 22.9	<0.001	0.168
Fatigue	57.8 ± 30.8	40.2 ± 28.6	30.4 ± 23.7	43.4 ± 30.1	<0.001	0.136
Nausea/vomiting	26.8 ± 30.3	20.5 ± 26.1	11.3 ± 26.2	20.0 ± 28.1	0.015	0.047
Pain	43.5 ± 35.7	35.4 ± 34.8	18.7 ± 27.5	33.3 ± 34.5	<0.001	0.083
Dyspnoea	22.6 ± 31.8	16.2 ± 28.2	4.7 ± 16.5	15.0 ± 27.6	0.003	0.067
Insomnia	50.8 ± 35.2	32.8 ± 33.3	30.7 ± 28.4	38.3 ± 33.7	0.002	0.072
Appetite loss	44.1 ± 37.9	34.8 ± 34.8	23.3 ± 33.2	34.7 ± 36.2	0.011	0.051
Constipation	39.5 ± 38.9	26.8 ± 33.2	21.3 ± 31.4	29.5 ± 35.4	0.019	0.045
Diarrhoea	19.8 ± 32.8	11.6 ± 24.5	6.7 ± 16.5	13.0 ± 26.2	0.029	0.040
Financial difficulties	48.0 ± 43.4	41.9 ± 43.1	25.3 ± 40.2	39.2 ± 43.1	0.018	0.045
Composite symptom burden	38.1 ± 22.9	27.3 ± 21.2	18.4 ± 16.0	28.4 ± 21.8	<0.001	0.129
HADS-Anxiety	9.0 ± 5.5	4.7 ± 3.9	2.9 ± 2.7	5.6 ± 4.9	<0.001	0.265
HADS-Depression	10.9 ± 4.6	7.3 ± 4.3	6.2 ± 4.3	8.2 ± 4.8	<0.001	0.168
HADS total	19.8 ± 8.7	12.1 ± 7.0	9.0 ± 5.9	13.8 ± 8.6	<0.001	0.273
RCI-10 total	18.3 ± 8.7	26.1 ± 8.6	31.8 ± 7.9	25.1 ± 10.0	<0.001	0.291
FACIT-Sp Meaning	11.6 ± 2.7	14.5 ± 1.5	15.8 ± 0.4	13.9 ± 2.5	<0.001	0.468
FACIT-Sp Peace	9.9 ± 2.9	13.2 ± 1.9	15.4 ± 0.9	12.7 ± 3.0	<0.001	0.521
FACIT-Sp Faith	9.8 ± 3.2	13.6 ± 2.3	15.8 ± 0.7	12.9 ± 3.4	<0.001	0.519

Data are mean ± standard deviation. *p* values are from one-way analysis of variance; η^2^ is the proportion of total variance explained by group. Because several symptom scales are skewed with floor effects, all comparisons were repeated with the Kruskal–Wallis test; the direction and significance of every row were unchanged (all Kruskal–Wallis *p* < 0.05). After Benjamini–Hochberg adjustment across the 25 comparisons, all remained significant at q < 0.05. EORTC functional and global scales are scored 0–100 with higher values indicating better status; symptom scales are scored 0–100 with higher values indicating greater burden. The RCI-10 total is the recorded 10-item score (possible range 10–50). Abbreviations: FACIT-Sp, Functional Assessment of Chronic Illness Therapy—Spiritual Well-Being; EORTC QLQ-C30, European Organisation for Research and Treatment of Cancer Quality of Life Questionnaire Core 30; HADS, Hospital Anxiety and Depression Scale; QoL, quality of life; RCI-10, Religious Commitment Inventory-10; SWB, spiritual well-being.

**Table 3:** Unadjusted and adjusted between-group differences in the primary and secondary outcomes, high versus low spiritual well-being.

Outcome	Low SWB	Intermediate SWB	High SWB	Crude Difference, High vs. Low (95% CI)	Adjusted Difference, High vs. Low (95% CI) ^a^	Hedges’ g (95% CI)	*p*, Crude	*p*, Adjusted
Global health status/QoL	53.8 ± 28.0	65.9 ± 22.3	74.5 ± 23.1	20.7 (11.0 to 30.4)	14.9 (4.3 to 25.5)	0.79 (0.40 to 1.19)	<0.001	0.006
Functional summary score	52.5 ± 22.4	68.1 ± 22.6	75.3 ± 16.6	22.9 (15.5 to 30.3)	16.5 (8.6 to 24.4)	1.14 (0.73 to 1.55)	<0.001	<0.001
Composite symptom burden	38.1 ± 22.9	27.3 ± 21.2	18.4 ± 16.0	−19.7 (−27.2 to −12.3)	−16.2 (−23.8 to −8.6)	−0.98 (−1.38 to −0.58)	<0.001	<0.001
Emotional functioning	57.5 ± 31.9	72.9 ± 25.2	86.8 ± 16.5	29.3 (19.9 to 38.8)	25.6 (15.3 to 35.9)	1.12 (0.72 to 1.53)	<0.001	<0.001
HADS-Anxiety	9.0 ± 5.5	4.7 ± 3.9	2.9 ± 2.7	−6.1 (−7.7 to −4.5)	−5.7 (−7.4 to −3.9)	−1.38 (−1.80 to −0.96)	<0.001	<0.001
HADS-Depression	10.9 ± 4.6	7.3 ± 4.3	6.2 ± 4.3	−4.7 (−6.4 to −3.0)	−4.1 (−5.9 to −2.3)	−1.04 (−1.44 to −0.63)	<0.001	<0.001

Group values are mean ± standard deviation. Crude differences and *p* values are from Welch’s t-test. ^a^ Adjusted for age, sex, advanced disease, ECOG performance status, comorbidity count and residence, using HC3 heteroscedasticity-consistent standard errors. Hedges’ g is the bias-corrected standardised mean difference. Abbreviations: CI, confidence interval; HADS, Hospital Anxiety and Depression Scale; QoL, quality of life; SWB, spiritual well-being.

**Table 4:** Exploratory subgroup analyses of the adjusted association between spiritual well-being and global quality of life, with tests for effect modification.

Subgroup	*n*	Adjusted β per +1 SD FACIT-Sp-12 (95% CI) ^a^	*p* Value	*p* for Interaction
Overall	175	7.18 (2.52 to 11.85)	0.003	
Female	89	8.97 (1.80 to 16.14)	0.014	0.620
Male	86	5.84 (−0.46 to 12.14)	0.069	0.620
Age < 65 years	75	6.75 (−3.15 to 16.65)	0.182	0.947
Age ≥ 65 years	100	8.02 (2.75 to 13.29)	0.003	0.947
Advanced disease	114	7.49 (1.28 to 13.70)	0.018	0.352
Non-advanced disease	61	5.38 (−1.93 to 12.69)	0.149	0.352
ECOG 0–1	113	9.13 (2.31 to 15.94)	0.009	0.630
ECOG 2–4	62	5.67 (−1.33 to 12.66)	0.112	0.630
Urban residence	100	9.53 (4.59 to 14.47)	<0.001	0.027
Rural residence	75	2.95 (−5.33 to 11.24)	0.485	0.027
On chemotherapy	130	5.76 (−0.04 to 11.57)	0.051	0.513
Not on chemotherapy	45	13.68 (3.92 to 23.43)	0.006	0.513
Attendance ≥ monthly	67	3.75 (−5.23 to 12.73)	0.414	0.416
Attendance < monthly	108	8.53 (3.20 to 13.85)	0.002	0.416
Sought spiritual support	127	11.69 (5.61 to 17.78)	<0.001	0.286
No spiritual support sought	48	1.27 (−5.51 to 8.05)	0.713	0.286

^a^ β is the change in EORTC QLQ-C30 global health status/quality of life (points, 0–100 scale) per one standard deviation increase in FACIT-Sp-12 total score, from linear models adjusted for age, sex, advanced disease and ECOG performance status with HC3 standard errors. *p* for interaction is from the multiplicative interaction term in the corresponding full-cohort model. Abbreviations: CI, confidence interval; ECOG, Eastern Cooperative Oncology Group; FACIT-Sp-12, Functional Assessment of Chronic Illness Therapy—Spiritual Well-Being 12-item scale; SD, standard deviation. Subgroup models omit comorbidity count to preserve degrees of freedom within strata, so the overall row differs trivially from the primary Model 1 estimate (7.15; 95% CI 3.45 to 10.86). *p* for interaction is unadjusted; after Benjamini–Hochberg adjustment across the eight interaction tests, no q value was below 0.05 (residence q = 0.22; all others q ≥ 0.63).

**Table 5:** Adjusted associations of each spirituality dimension with EORTC QLQ-C30 domains and psychological distress.

Outcome	Meaning, β per +1 SD (95% CI)	Peace, β per +1 SD (95% CI)	Faith, β per +1 SD (95% CI)	RCI-10, β per +1 SD (95% CI)
Global health status/QoL	5.90 (0.95 to 10.85); *p* = 0.020	7.73 (3.72 to 11.74); *p* < 0.001	3.44 (−0.43 to 7.31); *p* = 0.082	3.98 (0.07 to 7.88); *p* = 0.046
Physical functioning	7.31 (3.29 to 11.32); *p* < 0.001	8.69 (4.57 to 12.81); *p* < 0.001	1.23 (−2.64 to 5.10); *p* = 0.535	0.50 (−3.85 to 4.86); *p* = 0.820
Role functioning	4.87 (−1.70 to 11.45); *p* = 0.146	7.14 (1.68 to 12.60); *p* = 0.010	1.29 (−3.49 to 6.07); *p* = 0.598	0.45 (−4.41 to 5.32); *p* = 0.856
Emotional functioning	7.18 (1.10 to 13.26); *p* = 0.021	15.00 (10.26 to 19.73); *p* < 0.001	2.85 (−2.12 to 7.83); *p* = 0.261	1.68 (−3.00 to 6.35); *p* = 0.483
Cognitive functioning	7.61 (3.58 to 11.64); *p* < 0.001	10.30 (6.47 to 14.13); *p* < 0.001	2.18 (−1.35 to 5.70); *p* = 0.227	0.81 (−2.84 to 4.47); *p* = 0.663
Social functioning	1.83 (−3.79 to 7.46); *p* = 0.523	1.80 (−3.65 to 7.25); *p* = 0.517	1.00 (−4.48 to 6.49); *p* = 0.720	3.58 (−1.92 to 9.08); *p* = 0.202
Composite symptom burden	−5.92 (−9.93 to −1.92); *p* = 0.004	−7.04 (−10.40 to −3.67); *p* < 0.001	−1.09 (−3.95 to 1.76); *p* = 0.453	−2.15 (−5.13 to 0.84); *p* = 0.158
HADS-Anxiety	−1.79 (−2.55 to −1.03); *p* < 0.001	−3.24 (−3.88 to −2.61); *p* < 0.001	−1.01 (−1.84 to −0.19); *p* = 0.016	−0.72 (−1.44 to −0.00); *p* = 0.049
HADS-Depression	−1.70 (−2.43 to −0.96); *p* < 0.001	−2.19 (−2.90 to −1.48); *p* < 0.001	−1.04 (−1.78 to −0.29); *p* = 0.007	−0.62 (−1.38 to 0.14); *p* = 0.109

Each cell is from a separate linear model containing one spirituality predictor, adjusted for age, sex, advanced disease, ECOG performance status and comorbidity count, with HC3 standard errors. β is the change in the outcome per one standard deviation increase in the predictor. Abbreviations: CI, confidence interval; EORTC QLQ-C30, European Organisation for Research and Treatment of Cancer Quality of Life Questionnaire Core 30; HADS, Hospital Anxiety and Depression Scale; QoL, quality of life; RCI-10, Religious Commitment Inventory-10; SD, standard deviation.

**Table 6:** Spearman rank correlation matrix of spirituality, quality of life, distress and performance status measures.

Variable	1	2	3	4	5	6	7	8	9	10	11
1. FACIT-Sp-12 total	1										
2. Meaning	0.79 ***	1									
3. Peace	0.82 ***	0.63 ***	1								
4. Faith	0.78 ***	0.42 ***	0.39 ***	1							
5. RCI-10	0.56 ***	0.35 ***	0.26 ***	0.70 ***	1						
6. Global QoL	0.38 ***	0.40 ***	0.40 ***	0.20 **	0.21 **	1					
7. Functional summary	0.44 ***	0.45 ***	0.51 ***	0.18 *	0.15 *	0.61 ***	1				
8. Symptom burden	−0.41 ***	−0.43 ***	−0.46 ***	−0.15 *	−0.17 *	−0.59 ***	−0.72 ***	1			
9. HADS-A	−0.55 ***	−0.42 ***	−0.67 ***	−0.26 ***	−0.18 *	−0.43 ***	−0.54 ***	0.50 ***	1		
10. HADS-D	−0.47 ***	−0.42 ***	−0.53 ***	−0.23 **	−0.18 *	−0.43 ***	−0.50 ***	0.44 ***	0.60 ***	1	
11. ECOG	−0.30 ***	−0.33 ***	−0.34 ***	−0.17 *	−0.19 *	−0.46 ***	−0.60 ***	0.50 ***	0.32 ***	0.32 ***	1

Values are Spearman’s ρ. * *p* < 0.05; ** *p* < 0.01; *** *p* < 0.001. Column numbers correspond to the numbered row variables. Abbreviations: ECOG, Eastern Cooperative Oncology Group; FACIT-Sp-12, Functional Assessment of Chronic Illness Therapy—Spiritual Well-Being 12-item scale; HADS-A, Hospital Anxiety and Depression Scale—anxiety; HADS-D, Hospital Anxiety and Depression Scale—depression; QoL, quality of life; RCI-10, Religious Commitment Inventory-10.

**Table 7:** Multivariable linear regression model for EORTC QLQ-C30 global health status/quality of life.

Predictor	β	95% CI	Standardised β	*p* Value	VIF
Spiritual well-being (per +1 SD FACIT-Sp-12)	1.17	−4.10 to 6.44	0.045	0.664	1.76
Composite symptom burden (per +1 SD)	−7.85	−13.95 to −1.76	−0.304	0.012	1.63
HADS-Depression (per +1 point)	−0.91	−1.89 to 0.06	−0.170	0.067	1.62
HADS-Anxiety (per +1 point)	−0.76	−1.80 to 0.29	−0.143	0.157	1.94
Age (per +1 year)	−0.03	−0.37 to 0.32	−0.014	0.877	1.29
Male sex	2.68	−4.34 to 9.69	0.052	0.455	1.13
Advanced disease	0.66	−7.43 to 8.76	0.012	0.872	1.29
ECOG (per +1 category)	−4.86	−8.73 to −1.00	−0.221	0.014	1.73
Comorbidity count (per +1)	−0.15	−3.21 to 2.91	−0.008	0.925	1.42
Urban residence	−0.99	−7.83 to 5.84	−0.019	0.776	1.05
Time since diagnosis (per +1 month)	0.03	−0.05 to 0.11	0.064	0.399	1.07

Model fitted with HC3 heteroscedasticity-consistent standard errors; *n* = 168 complete cases; R^2^ = 0.431; adjusted R^2^ = 0.39; residual standard error = 20.38 points; F = 12.25, *p* < 0.001. β is the change in global quality of life (0–100 points) per unit of the predictor. Abbreviations: CI, confidence interval; ECOG, Eastern Cooperative Oncology Group; FACIT-Sp-12, Functional Assessment of Chronic Illness Therapy—Spiritual Well-Being 12-item scale; HADS, Hospital Anxiety and Depression Scale; SD, standard deviation; VIF, variance inflation factor.

**Table 8:** Bootstrapped mediation analysis of the pathways linking symptom burden, spiritual well-being, emotional distress and global quality of life.

Pathway	Path a (95% CI)	Path b (95% CI)	Total Effect c (95% CI)	Direct Effect c′ (95% CI)	Indirect Effect a × b (95% CI)	Proportion of Total Association (Descriptive)	*p*, Indirect ^a^
Symptom burden → spiritual well-being → global QoL	−0.276 (−0.427 to −0.125)	4.665 (1.075 to 8.256)	−10.78 (−14.39 to −7.17)	−9.50 (−13.18 to −5.81)	−1.29 (−3.08 to −0.20)	11.9%	0.013
Symptom burden → HADS-depression → global QoL	1.561 (0.812 to 2.309)	−1.290 (−2.000 to −0.581)	−10.78 (−14.39 to −7.17)	−8.77 (−12.43 to −5.11)	−2.01 (−3.75 to −0.61)	18.7%	0.004
Spiritual well-being → HADS-anxiety → global QoL	−2.545 (−3.218 to −1.871)	−1.655 (−2.457 to −0.854)	7.15 (3.45 to 10.86)	2.94 (−1.15 to 7.03)	4.21 (1.89 to 6.86)	58.9%	<0.001
Spiritual well-being → HADS-depression → global QoL	−2.068 (−2.765 to −1.371)	−1.467 (−2.248 to −0.687)	7.15 (3.45 to 10.86)	4.12 (0.20 to 8.04)	3.03 (1.17 to 5.43)	42.4%	<0.001
Spiritual well-being → HADS-anxiety + HADS-depression (parallel) → global QoL	−2.545 (anxiety); −2.068 (depression)	−1.02 (−1.97 to −0.07) (anxiety); −0.90 (−1.79 to −0.01) (depression)	7.15 (3.45 to 10.86)	2.69 (−1.42 to 6.80)	Total 4.46 (2.10 to 7.02); via anxiety 2.60 (0.22 to 5.10); via depression 1.86 (0.02 to 3.96)	62.4%	<0.001
FACIT-Sp peace → HADS-anxiety → global QoL	−3.243 (−3.823 to −2.663)	−1.623 (−2.538 to −0.708)	7.73 (4.13 to 11.33)	2.47 (−2.11 to 7.04)	5.26 (1.57 to 8.94)	68.1%	0.004

All paths adjusted for age, sex, advanced disease, ECOG performance status and comorbidity count. Exposures are expressed per one standard deviation; the spiritual well-being mediator per one standard deviation and the HADS mediators per one point; the outcome in points of global quality of life (0–100). ^a^ Two-sided *p* value for the indirect effect from 5000 non-parametric bootstrap resamples using the percentile method. The parallel-mediator row enters HADS-anxiety and HADS-depression simultaneously in the outcome model, so that the specific indirect associations are mutually adjusted; the single-mediator rows for anxiety and depression estimate overlapping pathways, and their proportions mediated are not additive. All indirect effects are cross-sectional statistical decompositions and do not establish temporal ordering; the proportion of the total association is the ratio of the indirect to the total coefficient and is reported as a descriptive quantity, not as the share of a causal effect. Abbreviations: CI, confidence interval; FACIT-Sp, Functional Assessment of Chronic Illness Therapy—Spiritual Well-Being; HADS, Hospital Anxiety and Depression Scale; QoL, quality of life.

**Table 9:** Sensitivity analyses of the adjusted association between spiritual well-being and global quality of life.

Analysis	*n*	β per +1 SD FACIT-Sp-12	95% CI	*p* Value
Model 2 reference (HC3 robust SE, complete case)	175	4.67	0.37 to 8.96	0.033
Ordinary least squares (model-based SE)	175	4.67	1.07 to 8.26	0.011
Excluding patients in remission	164	4.81	0.51 to 9.11	0.028
Restricted to ECOG 0–2	148	1.86	−2.96 to 6.68	0.449
Additionally adjusted for tumour site and education	175	5.08	0.51 to 9.64	0.029
Additionally adjusted for HADS anxiety and depression	175	1.47	−3.41 to 6.35	0.556
Median (quantile) regression	175	6.64	2.41 to 10.86	0.002
Multiple imputation for missing data (20 imputations; extended Model 2)	175	4.55	0.98 to 8.11	0.012
Non-parametric bootstrap (2000 resamples)	175	4.83	1.08 to 8.88	0.011
Inverse-probability-of-treatment weighting (high vs. low SWB)	175	14.00	6.52 to 21.47	<0.001

Unless stated otherwise, models are adjusted for age, sex, advanced disease, ECOG performance status and comorbidity count, in addition to composite symptom burden. The inverse-probability-of-treatment-weighting row contrasts the upper against the lower half of the FACIT-Sp-12 distribution and is therefore on a different scale from the other rows. The Model 2 reference estimate is a sensitivity analysis that conditions on symptom burden and is not the primary estimand (Model 1: 7.15; 95% CI 3.45 to 10.86). The multiple-imputation row is fitted to the extended Model 2 (additionally including residence and time since diagnosis), the only specification in which missing values occur; its complete-case counterpart (*n* = 168) gave 4.49 (95% CI 0.12 to 8.86; *p* = 0.044), so the difference from the reference row reflects the two additional covariates rather than imputation. In the inverse-probability-weighting row, the propensity model, weights and weighted contrast were estimated within each of the 20 imputed datasets and pooled by Rubin’s rules (*n* = 175); the complete-case weighted contrast (*n* = 168) was 13.6 (95% CI 5.9 to 21.3; *p* < 0.001). The sample, covariates and missing-data handling of each row are listed in Table 10. Inverse-probability weighting diagnostics (averaged across the imputed datasets; propensity-model covariates in Table 10): full propensity-score overlap (0.14–0.83 in both groups); stabilised weights median 0.98 (0.41–2.62 after truncation); effective sample size 153; largest absolute standardised mean difference 0.31 before and 0.07 after weighting (all covariates < 0.10); robust sandwich variance. Abbreviations: CI, confidence interval; ECOG, Eastern Cooperative Oncology Group; FACIT-Sp-12, Functional Assessment of Chronic Illness Therapy—Spiritual Well-Being 12-item scale; SD, standard deviation.

**Table 10:** Analytic sample, model covariates and missing-data handling for each sensitivity analysis in Table 9.

Analysis	*n*	Covariates in the Outcome Model	Missing-Data Handling
Model 2 reference (HC3 robust SE, complete case)	175	Age, sex, advanced disease, ECOG performance status, comorbidity count, composite symptom burden	None required: no missing values in any model variable
Ordinary least squares (model-based SE)	175	As Model 2	None required
Excluding patients in remission	164	As Model 2	None required
Restricted to ECOG 0–2	148	As Model 2	None required
Additionally adjusted for tumour site and education	175	Model 2 plus primary tumour site (seven categories) and education (four categories)	None required
Additionally adjusted for HADS anxiety and depression	175	Model 2 plus HADS-anxiety and HADS-depression	None required
Median (quantile) regression	175	As Model 2	None required
Multiple imputation for missing data (20 imputations)	175	Extended Model 2: Model 2 plus residence and time since diagnosis	Time since diagnosis (*n* = 7) and role functioning (*n* = 1) imputed by chained equations (predictive mean matching); imputation model contained all analysis variables and the outcome; estimates pooled by Rubin’s rules. Complete-case counterpart: *n* = 168, β = 4.49 (95% CI 0.12 to 8.86)
Non-parametric bootstrap (2000 resamples)	175	As Model 2	None required
Inverse-probability-of-treatment weighting (high vs. low SWB)	175	Weighted outcome model containing the exposure indicator only; propensity model: age, sex, advanced disease, ECOG performance status, comorbidity count, residence, time since diagnosis	Propensity model, weights and weighted contrast estimated within each of the 20 imputed datasets and pooled by Rubin’s rules. Complete-case weighted contrast: *n* = 168, 13.6 (95% CI 5.9 to 21.3)

Missing values occurred only for time since diagnosis (*n* = 7) and role functioning (*n* = 1); role functioning enters the functional summary score (a secondary outcome) and is not used in any model in Table 9. The primary Model 1 and the Model 2 reference therefore contain no missing values and are complete-case by construction. Abbreviations: CI, confidence interval; ECOG, Eastern Cooperative Oncology Group; HADS, Hospital Anxiety and Depression Scale; HC3, heteroscedasticity-consistent (type 3) standard errors; SE, standard error; SWB, spiritual well-being.

## Data Availability

The data presented in this study are available on reasonable request from the corresponding author. The data are not publicly available because they contain information that could compromise the privacy of research participants.
